# CXCR1 and CXCR2 Inhibition by Ladarixin Improves Neutrophil-Dependent Airway Inflammation in Mice

**DOI:** 10.3389/fimmu.2020.566953

**Published:** 2020-10-02

**Authors:** Matheus Silverio Mattos, Maximiliano Ruben Ferrero, Lucas Kraemer, Gabriel Augusto Oliveira Lopes, Diego Carlos Reis, Geovanni Dantas Cassali, Fabricio Marcus Silva Oliveira, Laura Brandolini, Marcello Allegretti, Cristiana Couto Garcia, Marco Aurélio Martins, Mauro Martins Teixeira, Remo Castro Russo

**Affiliations:** ^1^ Laboratory of Comparative Pathology, Department of Physiology and Biophysics, Institute of Biological Sciences, Universidade Federal de Minas Gerais, Belo Horizonte, Brazil; ^2^ Laboratory of Inflammation, Oswaldo Cruz Institute, Fiocruz, Rio de Janeiro, Brazil; ^3^ Laboratory of Comparative Pathology, Department of General Pathology, Institute of Biological Sciences, Universidade Federal de Minas Gerais, Belo Horizonte, Brazil; ^4^ R&D Department, Dompé Farmaceutici S.p.a., L’Aquila, Italy; ^5^ Laboratory of Respiratory Virus and Measles, Instituto Oswaldo Cruz, Fiocruz, Rio de Janeiro, Brazil; ^6^ Laboratory of Immunopharmacology, Department of Biochemistry and Immunology, Institute of Biological Sciences, Universidade Federal de Minas Gerais, Belo Horizonte, Brazil

**Keywords:** neutrophils (PMNs), asthma, fibrosis, chronic obstructive pulmonary disease, influenza A (H1N1)

## Abstract

**Rationale:**

Increased IL-8 levels and neutrophil accumulation in the airways are common features found in patients affected by pulmonary diseases such as Asthma, Idiopathic Pulmonary Fibrosis, Influenza-A infection and COPD. Chronic neutrophilic inflammation is usually corticosteroid insensitive and may be relevant in the progression of those diseases.

**Objective:**

To explore the role of Ladarixin, a dual CXCR1/2 antagonist, in several mouse models of airway inflammation with a significant neutrophilic component.

**Findings:**

Ladarixin was able to reduce the acute and chronic neutrophilic influx, also attenuating the Th2 eosinophil-dominated airway inflammation, tissue remodeling and airway hyperresponsiveness. Correspondingly, Ladarixin decreased bleomycin-induced neutrophilic inflammation and collagen deposition, as well as attenuated the corticosteroid resistant Th17 neutrophil-dominated airway inflammation and hyperresponsiveness, restoring corticosteroid sensitivity. Finally, Ladarixin reduced neutrophilic airway inflammation during cigarette smoke-induced corticosteroid resistant exacerbation of Influenza-A infection, improving lung function and mice survival.

**Conclusion:**

CXCR1/2 antagonist Ladarixin offers a new strategy for therapeutic treatment of acute and chronic neutrophilic airway inflammation, even in the context of corticosteroid-insensitivity.

**Graphical Abstract:**
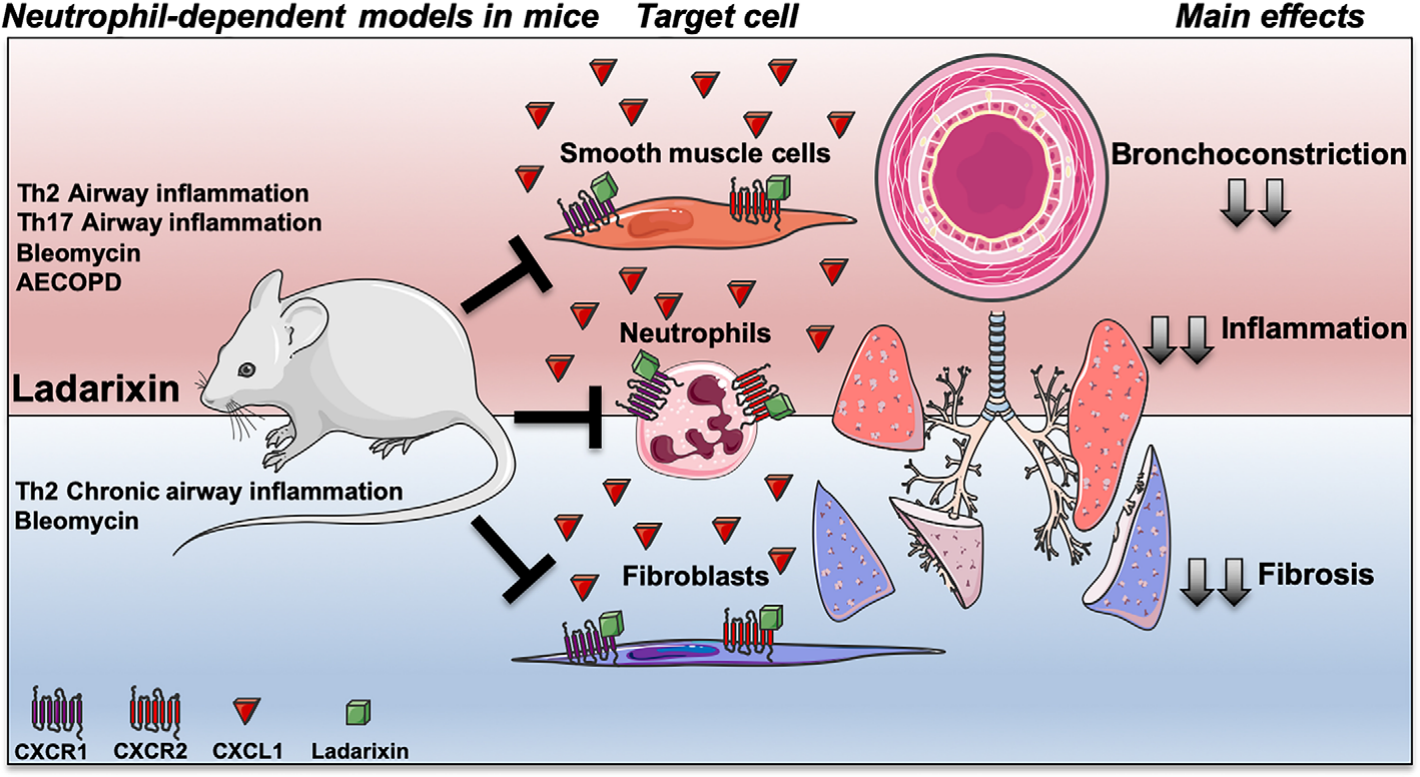
Targeting CXCR1 and CXCR2 inhibition by Ladarixin improves neutrophil-dependent airway inflammation in mice. Ladarixin, a non-competitive allosteric CXCR1 and CXCR2 antagonist, emerges as an efficacious candidate to treat a broad range of neutrophilic-mediated respiratory diseases, from asthma (Th2 or Th17-skewed models) to cigarette smoke-induced exacerbation of Influenza-A infection (AECOPD). The oral treatment with Ladarixin reduced the main manifestations of disease in acute models (red) of asthma and AECOPD, and in chronic models (blue) of asthma and fibrosis. The inhibition of CXCR1 and CXCR2 by Ladarixin importantly has anti-inflammatory effect mainly by reducing neutrophil recruitment into the lungs mimicking the GC in context of steroid-sensitive asthma models (Th2-skewed), but also in context of Steroid-resistance in Glucocorticoid (GC)-refractory asthma (Th17-skewed) and AECOPD models. Finally, Ladarixin may act in pulmonary resident cell, directly on CXCR1 and CXCR2 expressed by airway smooth muscle cell improving airway bronchoconstriction, as well as, it can reduce collagen deposition by blocking CXCR1 and CXCR2 expressed by lung resident fibroblasts.

## Introduction

Chemokines are small molecules from the cytokine family, which play a crucial physiological role in the biology of leukocytes and tissue resident cells (1). The chemokine receptors CXCR1 and CXCR2 are G protein-coupled receptors that bind to the human IL-8 family chemokines (CXCL1, CXCL2, CXCL3, CXCL5, CXCL6, CXCL7 and CXCL8) and were first described by their ability to promote neutrophil migration (1). CXCR1 and CXCR2 are also found on NK cells (2) and lung structural cells (3), such as fibroblasts, epithelial and endothelial cells. There is a growing body of evidence indicating that CXCR1 and CXCR2 may have a role in the pathogenesis of certain pulmonary diseases, including COPD, lung fibrosis and asthma (3). Moreover, the common point between these various diseases is the chronic presence of neutrophils in the lungs, generally associated with a high level of ELR+ chemokines and expression of CXCR1 and CXCR2 receptors. In this context, it is believed that CXCR1 and CXCR2 could be potential targets to restrain the immune response during respiratory allergies, pulmonary remodeling and scaring, lung infection, smoke exposure and inflammatory exacerbations (1).

Asthma is a chronic inflammatory allergic airway disorder that causes variable airflow obstruction in association with airway hyperreactivity (AHR) and mucus overproduction (4), affecting up to 300 million people worldwide (5). Most asthma patients have mild to moderate disease that respond adequately to inhaled glucocorticoids (GCs) in combination or not with β2-adrenergic bronchodilators (6). Approximately 10% of asthma patients respond poorly to GCs and these patients often present the severe form of the disease (6–8). In patients with the severe form of asthma, there is a positive correlation between increased levels of ELR+ CXC chemokines and the presence of neutrophils in lung tissue and BALF (9, 10). Experiments using various animal models of asthma have suggested the relevance of CXCR2 and its ligands in the pathogenesis of the disease (11–13). Moreover, increased levels of CXCL8 in BALF have been found in patients with severe asthma refractory to glucocorticoids (14). Idiopathic pulmonary fibrosis (IPF) is a chronic disease characterized by an excessive fibrogenesis and immune system activation, with increased extracellular matrix deposition and irreversible organ dysfunction, in response to alveolar epithelium injury (15) related to significant leukocyte migration into the airways. IPF patients had increased CXCL8 levels in the airways, which were associated with neutrophilic infiltration and disease severity (16, 17). There is evidence showing the participation of CXCR1 and CXCR2 (18–20) and ligands (21) in the development of experimental pulmonary fibrosis. Therefore, the CXCR1 and CXCR2 activation may contribute to the pulmonary fibrosis in human and mouse models. Chronic obstructive pulmonary disease (COPD) is primarily associated with cigarette smoke (Cs) exposure that leads to sustained lung inflammation and proteolysis of extracellular matrix (22). COPD patients frequently experience acute exacerbations of their symptoms (AECOPD), mostly associated to viral or bacterial infections, which enhance lung neutrophil inflammation and are also accompanied by significant morbidity and mortality (22–24). During AECOPD increased number of neutrophils migrate to the airways in response to CXCL5, CXCL8, and CXCR2 expression in lungs (25). Notably, GCs fail to avoid disease progression or exacerbations, indicating that novel safer and more effective treatments to control AECOPD are needed.

In this scenario, several pharmaceutical companies have drawn their attention to identify CXCR1 and CXCR2 chemokine receptors antagonists to treat pulmonary diseases (1, 3). Particularly, Dompè pharma has developed a selective dual CXCR1 and CXCR2 antagonist Ladarixin, previously named DF2156A (18). Ladarixin is an allosteric non-competitive antagonist, orally available, currently tested in Phase 2 clinical trials (NCT02814838, NCT01571895). Thus, the aim of this work was to investigate the effects of Ladarixin in the context of a range of pulmonary inflammatory diseases with contribution or predominance of neutrophils. Here we report that Ladarixin was able to reduce inflammation and ameliorate lung function in both acute Th2-predominant allergic airway inflammation and Th17-predominant neutrophilic inflammation in a GC-refractory asthma model. Ladarixin also decreased bleomycin-induced neutrophilic inflammation and collagen deposition, and controlled neutrophilic airway inflammation during a GC resistant model of cigarette smoke-induced exacerbation of Influenza-A infection, enhancing lung function and improving survival. Thus, CXCR1 and CXCR2 inhibition might have an important therapeutic contribution in a broad range of pulmonary pathologies with a significant neutrophilic component.

## Methods

### Mice

Male Balb/c mice and ΔdblGATA-1 knockout mice (26), 8-10 weeks old were obtained from the Central Animal Facility from the Federal University of Minas Gerais, Brazil. Female C57/BL6 mice, 8-10 weeks old were obtained from Institute of Sciences and Technology in Biomodels of the Oswaldo Cruz Institute, Fiocruz, Brazil. Mice were housed in ventilated cages in a controlled day-night cycle and had access to food and water *ad libitum.* All experiments were analyzed and approved by the local ethics committee: Steroid-sensitive and insensitive allergic airway inflammation models induced by ovalbumin, Ethical committee CEUA/UFMG number 88/2015; Bleomycin-induced pulmonary inflammation and fibrosis model, Ethical committee CEUA/UFMG number 172/14; EADPOC model, Ethical committee CEUA/IOC number L-030/15.

### Th2 Acute Asthma Model

Th2 acute asthma model was performed as previously described (27, 28). Briefly, the classic Th2 GC-sensitive OVA/Alum model, BALB/c mice received systemic immunization by subcutaneous (*s.c.*) injection of 10 µg of chicken egg ovalbumin (OVA grade V, >98% pure; Sigma, St Louis, MO) diluted in 2 mg/ml of Al(OH)3 followed by a booster injection at the day 14. Nasal challenges were performed starting at the day 28, by inhalational exposure to aerosolized ovalbumin in an acrylic box for 20 min per day during 4 consecutive days. A solution of 1% ovalbumin in saline 0,9% was aerosolized by delivery of compressed air to a side stream jet nebulizer (OMRON HEALTHCARE Co., Ltd. NE-U700).

We also performed a single-OVA exposure model of Th2 acute asthma model as previously described (29). In these experiments, mice were immunized as mentioned above and challenged with a single-OVA nebulization for 20 min at the day 28. Pulmonary mechanics and BALF analysis were performed 2 or 6 h after the OVA challenge. These experiments were designed to further investigate the early-phase response in asthma, the kinetics of cytokines/chemokines production and leukocytes migration into the airways as well as the onset of physiological changes in the lungs.

### Th2 Chronic Asthma Model

Th2 chronic asthma model was performed as we previously described (27). Briefly, on days 1 and 14, mice were immunized by *s.c.* injection of 10 µg of OVA diluted in 2 mg/ml Alum. Nasal challenges were performed starting at the day 28 by inhalational exposure to aerosolized ovalbumin 1% in saline for 20 min per day, on 16 alternated days.

### Th17 GC-Insensitive Asthma Model

Th17 GC-insensitive asthma model was performed as previously described (27, 30). The GC-insensitive OVA/CFA model, BALB/c mice received systemic immunization by subcutaneous injection of 20 µg of chicken egg ovalbumin (OVA grade V, >98% pure; Sigma, St Louis, MO) in saline, emulsified in 75 µl CFA (Sigma-Aldrich). On days 28-31 mice were exposed to aerosolized ovalbumin (1% in saline) for 20 min per day.

### Bleomycin-Induced Pulmonary Inflammation and Fibrosis Model

Bleomycin-induced pulmonary inflammation and fibrosis model was performed as previously described (20). Mice were anesthetized with 80-µl *i.p.* of ketamine and xylazine (130 mg/kg ketamine and 8.5 mg/kg xylazine) solution, and instilled with 40 µl of 6.25 mg/kg of bleomycin (Blenoxane; Bristol-Meyers) in PBS, or PBS only in control group.

### Cigarette Smoke-Induced Exacerbation of Influenza-A Infection Model

Female C57BL/6 mice weighting 18-20 grams with 8-10 weeks old were exposed to cigarette smoke (Cs) from 12 commercial full-flavored Marlboro cigarettes (10 mg tar, 0.9 mg nicotine, and 10 mg monoxide) per day or ambient air (controls) for 11 days, using a smoking chamber as described previously (31). On the 7th day of exposure, the animals were anesthetized with 50-µl *s.c.* of ketamine (60 mg/kg) and xylazine (4 mg/kg) solution and infected with 1,000 pfu of influenza (Flu) virus strain A/PR/8/34 H1N1 intranasally. On day 10 or 12 of Cs exposure mice were euthanized with 100-µl *i.p.* of Ketamine (300 mg/kg) and Xylazine (30 mg/kg) solution and experiments were performed to evaluate lung inflammation. Alternatively, the same groups were evaluated for 14 days after H1N1 infection for lethality assessment. Groups receiving only viral infection (Flu), Cs (Cs) or no insult (Air) were also included as controls.

### Pharmacological Schedule

In this work we utilized Ladarixin, a selective dual CXCR1 and CXCR2 non-competitive allosteric antagonist, formerly known as DF2156A (18) (Dompé Farmaceutici SpA, L’Aquila AQ, Italy) and Dexamethasone, a steroidal anti-inflammatory drug. Mice were treated daily, 60 min before OVA challenge, with Ladarixin or dexamethasone starting at day 28 after first immunization. Ladarixin was administered orally by gavage (10 mg/kg in carboxymethylcellulose 0.5%) once a day. This Ladarixin dosage was chosen based on previous studies that described Ladarixin pharmacokinetics, pharmacodynamics and pharmacological characterization of this compound (18, 32). Dexamethasone (5 mg/kg in saline) was given subcutaneously twice a day. In the cigarette smoke–induced exacerbation of Influenza-A infection model, Ladarixin was administered orally by gavage (10 mg/kg in saline solution) once a day for 3 days starting at day 9, 48 h after viral infection, 60 min before the first Cs exposure of the day.

### Assessment of Leukocyte Content in Airways

To assess the leukocytes in airways, bronchoalveolar lavage fluid (BALF) was performed as we previously described (20, 27). Briefly, an incision was made in the trachea where a 1.7mm outside diameter polyethylene catheter was inserted. Then, the airways were washed 2 times with 1 ml of phosphate buffer saline (PBS). The samples were centrifuged at 300 x g for 10 min at 4°C, to the pellet was added 100 μl of 3% BSA in PBS and a sample from this suspension was used to assess the total leukocytes in the airways. The remaining was centrifuged at 300 x g for 10 min at 4°C on a histology glass slide and stained with Diff Quick (MICROPTIC). The differential count was performed based on morphology of the cells, to assess the percentage of macrophages, lymphocytes, eosinophils and neutrophils over the total cell number. The analysis was performed using an optical microscopy. Total protein was quantified in the BALF supernatant using a Bio-Rad protein assay Dye Reagent (Bio-Rad, USA) to measure possible protein leakage previously described (27).

## Chemokines, Cytokines, and Immunoglobulins Analysis

Levels of chemokines, cytokines and immunoglobulins were quantified as previously described (27, 28). Briefly, after BALF, right lung was removed and a sample of 100 mg was homogenized in 1 ml of PBS (0.4 M NaCl and 10 mM of NaPO4) containing anti-proteases (0.1 mM phenylmethylsulfonyl fluoride, 0.1 mM benzetonium chloride, 10 mM EDTA and 20 KI aprotinin (A) The homogenate was centrifuged 8,000 x g for 10 min at 4°C. The supernatant was frozen for further ELISA assay and the pellet was frozen/thawed three times using liquid nitrogen to access neutrophil myeloperoxidase (MPO) and eosinophil peroxidase (EPO) activities. Levels of IL-4, IL-5, IL-6, IL-13, IL-17A, TGF-β1, CXCL1/KC, CCL2/MCP-1, and CCL11/Eotaxin in lung tissue or serum, and titration of anti-OVA antibodies IgA in BAL, and serum IgG and IgE levels measured by ELISA technique using commercial DuoSet kits from R&D Systems, according to the instructions of the manufacturer. Results were expressed in levels of cytokines per ml of lung lysate (pg/ml), arbitrary units (A.U.) or optical density (O.D.).

### Neutrophil and Eosinophil Peroxidase Activities Assay

The neutrophil myeloperoxidase (MPO) and eosinophil peroxidase (EPO) activities were measured as previously described (27). MPO assay was performed in a 96-well microplate by adding, in each well, 25 µl of 3,3′-5,5′-tetramethylbenzidine (TMB Sigma) diluted in dimethyl sulfoxide (DMSO; Merck, Darmstadt, Germany) at a final concentration of 1.6 mM; 100 µl of H2O2 0.02% vol/vol in PBS (pH 5.4) containing HTAB and 25 µl of processed tissue supernatant. The reaction started, at 37 °C, by adding the TMB solution and the supernatant to the microplate and, after 5 min, H2O2 was added followed by a new incubation at 37 °C for 5 min. The reaction was stopped by adding 100 µl of H2SO4 1M and quantified at 450 nm wavelengths in a spectrophotometer (VERSA max, Molecular Devices, CA). For the EPO assay, the supernatant of processed samples was distributed in 96-wells microplate followed by addition of 75 µl of OPD (1.5 mM in Tris-HCl 0.05 M pH 8.0 supplemented with 6.6 mM H_2_O_2_). After 30 min of incubation at room temperature, the reaction was stopped by adding 50 µl of H_2_SO_4_ 1 M and quantified at 492 nm in spectrophotometer, as previously described (27, 28).

### Assessment of Pulmonary Mechanics

Pulmonary dysfunction was measured as we previously described (27, 33, 34). For invasive *in vivo* assessment, mice were anesthetized and tracheostomized, then were placed in a whole-body plethysmograph to maintain spontaneous breathing connected to a computer-controlled ventilator (Forced Pulmonary Maneuver System^®^, Buxco Research Systems^©^, Wilmington, North Carolina USA). Under mechanical respiration the Dynamic Compliance (Cdyn) and Lung Resistance (Rl) were determined by Resistance and Compliance RC test. To measure the Total Lung Capacity (TLC) and Residual Volume (RV), Pressure-Volume maneuvers was performed, which inflates the lungs to a standard pressure of +30 cm H2O and then slowly exhales until a negative pressure of -30 cm H2O is reached. To measure the Forced Expiratory Volume (FEV) and Flow-volume curve, Fast-Flow Volume maneuvers were performed and Flow-Volume curve were recorded during this maneuver. To evaluate AHR, the same mice used in previous maneuvers (basal condition) received Methacholine, 1 mg/kg (Acetyl-β-methylcholine chloride, A-2251, Sigma-Aldrich St. Louis, MO, USA) *i.v.* and after 10 s, a new set of maneuvers were conducted to assess Rl changes. Non-invasive *in vivo* assessment of respiratory frequency and Penh was performed using barometric whole-body plethysmography (Buxco Research System, Wilmington, NC, USA) as we previously described (34). Measurements were recorded for 5 min for each animal in conscious, spontaneously breathing mice. Penh and respiratory frequency values were obtained before the first Cs exposure, and on days 7, 9, 10, 11 and 12 of Cs exposure.

### Collagen Quantification in Lungs

Hydroxyproline was measured to access the collagen content in the lung tissue as we previously described (19, 20, 27). Fragments weighting 100 mg of lungs were homogenized in 0.2% saline, frozen and lyophilized. Then, lyophilized tissue was subjected to alkaline hydrolysis in 75 µl of NaOH 10 M in 300 µl of H2O at 120°C for 20 min. An aliquot of 50 µl was then added to 450 µl of oxidizing reagent (0.056M chloramine T and n-propanol 10% in acetate-citrate buffer pH 6.5) for 20 min. Hydroxyproline standard curve was prepared likewise. After 20 min of reaction, 500 µl of 1 M p-dimethylaminebenzaldehyde diluted in n-propanol-perchloric acid (2:1 vol/vol) was added. The absorbance was quantified in a spectrophotometer (VERSAmax, Molecular devices) at 550 nm.

### Malondialdehyde (MDA) Quantification

MDA level, as a parameter of lipid peroxidation, was determined using the thiobarbituric acid reactive substances (TBARS) method (35). Lung tissue samples (100 μl) were mixed in 100 μl of 10% trichloroacetic acid and centrifuged for 15 min at 3,600 g at 4°C. Then, the supernatant (150 μl) was collected, and 150 μl of thiobarbituric acid was added. Samples were heated at 95°C for 10 min. MDA levels were determined by absorbance (532 nm) (SpectraMax M5, Molecular Devices) and expressed as nm/mg protein.

### Histological Analysis

After BALF, left lung was removed and fixe6d in formalin 4% in PBS (pH 7.4), as previously described. The tissue was dehydrated gradually in ethanol, embedded in paraffin, and cut into 4-μm sections and stained with H&E (27, 36), periodic acid Schiff (PAS) (27, 28) or Gomori’s trichrome (27, 36). Photomicrographs were obtained at 400x magnification and were analyzed using the software Image Pro Plus (Image-Pro Plus 4.1, Media Cybernetics, USA) under light microscopy. Nine to twelve bronchial areas per lung had their PAS or Gomori’s trichrome content quantified and the results were expressed as positive area (pixels) (27, 28, 36). Fibrosis was determined using Gomori’s trichrome stained slices, as previously described (27, 36). Semiquantitative histopathology analyses of inflammation were performed as described by (27). Briefly, ten random images per lung were acquired at 200x and pulmonary inflammation score was recorded according to a six grade scale where “0” corresponded to less than 1% of lung tissue area inflamed, “1” where up to 9% of lung area was affected by inflammation, “2” when 10% to 29% of lung tissue area was affected, “3” if 30% to 49% of lung tissue area was affected, “4” if 50% to 69% of lung tissue area was affected and “5” if more than 70% of the lung tissue area was affected by inflammation. Hemorrhage score was based on a four-degree scale where “0” was absence of hemorrhage, “1” was little areas with hemorrhage, 2 several areas presented moderate hemorrhage and “3” if there were several areas with intense hemorrhage.

### Statistical Analysis

All results were expressed as mean *±* SEM. Normalized data were analyzed by One-Way ANOVA with Tukey post-test, using the software GraphPad Prism 7.0. Differences were considered significant at P< 0.05.

## Results

### Early Neutrophil Influx and Bronchial Hyperreactivity Occurs Independently of Eosinophils in a Model of Single OVA Exposure in Mice

**Figure 1:**
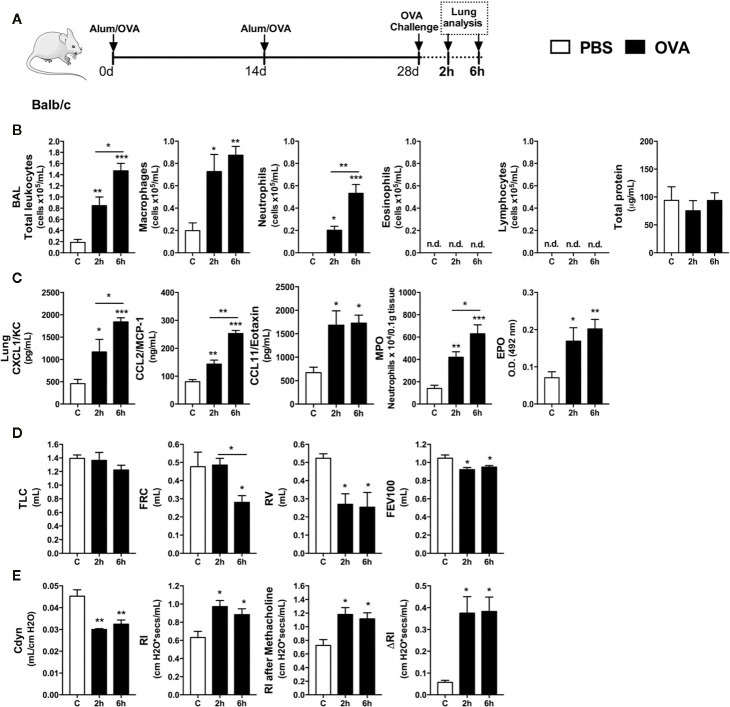
Characterization of early inflammatory infiltrate and pulmonary mechanics. Inflammatory parameters were evaluated 2 and 6 h after a single challenge with OVA. **(A)** Schematic representation of the experimental design. **(B)** Assessment of leukocytes influx and blood leakage into airways. **(C)** Chemokine levels, MPO and EPO activity assay in lung tissue. Assessment of pulmonary mechanics for **(D)** lung volumes, **(E)** compliance and lung resistance. n = 8 for each group, * for P < 0.05 ** for P < 0.01 *** for P < 0.001. TLC, total lung capacity; RV, residual volume; FRC, functional residual capacity; FEV100, forced expiratory volume in 100 ms; Cdyn, dynamic compliance; Rl, lung resistance; ΔRl, methacholine-evoked hyperresponsiveness.

Although airway eosinophilia is a hallmark of mild-to-moderated Th2 asthma, it is still elusive the kinetics of leukocytes recruitment into the airways. This first set of experiments was designed to investigated the first leukocyte groups that were recruited into the airways after single OVA challenge to OVA-immunized mice (Th2 asthma model - Alum/OVA). Leukocyte influx and cytokines were evaluated 2 and 6 h after challenge, as depicted in protocol schedule **(** **Figure 1A** **)**. There was an early influx of leukocytes in BALF, consisting mainly of macrophages and neutrophils, without eosinophils, lymphocytes or protein leakage in the alveolar space **(** **Figure 1B** **).** Moreover, we found an acute and progressive production of CXCL1, CCL2 and CCL11 in lung samples, which correlated with elevations in MPO and EPO activity **(** **Figure 1C** **)**. Besides the early neutrophil influx into the airways after OVA challenge, there was an indication of pulmonary dysfunction marked by decreased lung volumes, attested by TLC, RV, FRC, and FEV100 values **(** **Figure 1D** **)**, with loss of pulmonary elasticity assessed by reduced compliance (Cdyn), increasing in lung resistance (Rl) and bronchial hyperreactivity evoked by methacholine (ΔRl) **(** **Figure 1E** **)**. These results indicated that neutrophils are among the first leukocytes to be recruited into the airways after a single OVA challenge during the Th2 mice asthma model, and this early neutrophilic infiltration is accompanied by altered bronchial reactivity in mice.

**Figure 2:**
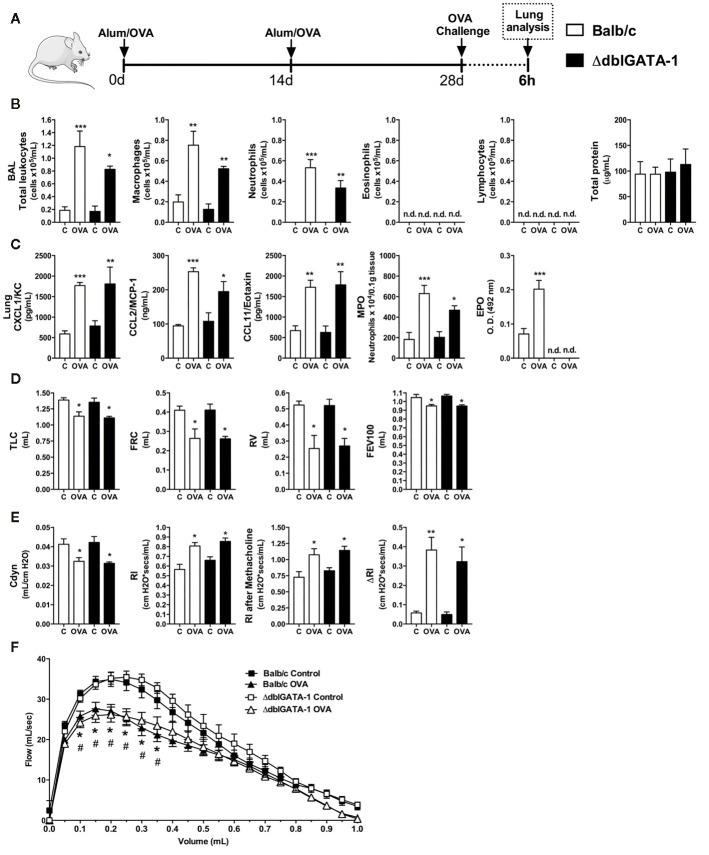
Eosinophils have minor role in the initial moments of Th2 allergy induced by OVA. Inflammatory parameters were evaluated 6 h after a single challenge with OVA. **(A)** Schematic representation of the experimental design. **(B)** Assessment of leukocytes influx and blood leakage into airways. **(C)** Chemokine levels, MPO and EPO activity assay in lung tissue. Assessment of pulmonary mechanics for **(D)** lung volumes, **(E)** compliance and lung resistance. **(F)** Flow-Volume curve. n = 8 for each group, * for P < 0.05; ** for P < 0.01 and *** for p < 0.001. TLC, total lung capacity; RV, residual volume; FRC, functional residual capacity; FEV100, forced expiratory volume in 100ms; Cdyn: Dynamic compliance; Rl: lung resistance; ΔRl, methacholine-evoked hyperresponsiveness. * differences between BALB/c control vs OVA. ^#^ differences between dblGATA1 control vs dblGATA1 OVA.

The presence of eosinophils in the airways and lungs is a hallmark of asthma. Due to the importance of these cells in the pathophysiology of classical Th2 asthma, we sought to investigate whether absence of eosinophils would impact the initial moments after OVA-induced allergic airway inflammation in mice. WT (Balb/c) or ΔdblGATA-1 knockout mice were immunized and challenged with OVA to evaluate leukocyte and cytokine profile at 6 h after OVA nebulization, as depicted in the protocol schedule **(** **Figure 2A** **)**. Both WT and eosinophil-deficient mice presented an increase in total leukocyte numbers in BALF markedly by macrophages and neutrophil influx, without lymphocytes and eosinophils influx, and protein leakage in airways **(** **Figure 2B** **)**. There were increased levels of CXCL1, CCL2, and CCL11 in lungs from WT and ΔdblGATA-1 knockout mice. There was also slightly increased MPO in both groups, and increased EPO activity in WT mice **(** **Figure 2C** **)**. Pulmonary function was impaired equally in WT and ΔdblGATA-1 KO mice, related to airway volumes loss (TLC, FRC, RV, and FEV100) **(** **Figure 2D** **)** with loss of elasticity (reduced compliance and increased resistance) with same airway hyperreactivity evoked by methacholine (ΔRl) (**Figure 2E** **),** and reduced airway flow, as shown by Flow x Volume curve **(** **Figure 2F** **)**. Collectively, these data indicate that eosinophils have a minor role in the initial development of Th2 allergic airway inflammation, pulmonary dysfunction and airway hyperreactivity in mice that could be related instead to early neutrophil influx.

### Ladarixin Reduced Allergic Airway Inflammation in a Model of Single OVA Exposure

**Figure 3:**
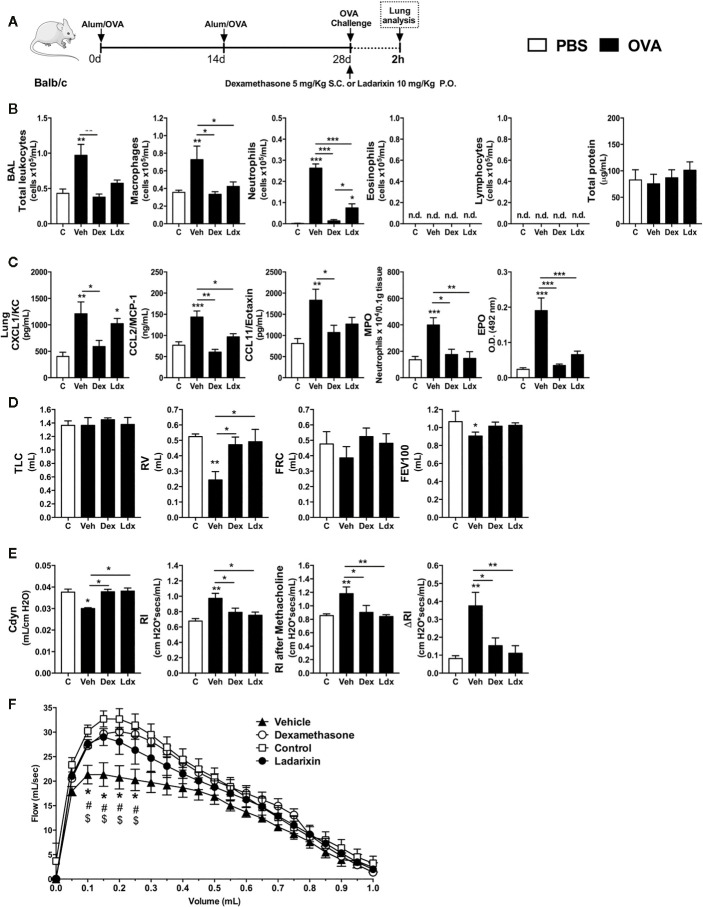
Ladarixin treatment reduces Th2 allergic airway inflammation induced by a single OVA challenge. Inflammatory parameters were evaluated 2 h after a single challenge with OVA. **(A)** Schematic representation of the experimental design. **(B)** Assessment of leukocyte influx and blood leakage into airways. **(C)** Chemokine levels, MPO and EPO activity assay in lung tissue. Assessment of pulmonary mechanics for **(D)** lung volumes, **(E)** compliance and lung resistance. **(F)** Flow-Volume curve. n = 8 for each group, * for P < 0.05; ** for P < 0.01 and *** for p < 0.001. TLC, total lung capacity; RV, residual volume; FRC, functional residual capacity; FEV100, forced expiratory volume in 100 ms; Cdyn, dynamic compliance; Rl, lung resistance; ΔRl, methacholine-evoked hyperresponsiveness. ^#^ differences between Dexamethasone vs Vehicle. ^$^ differences between Ladarixin vs Vehicle.

Once we detected that neutrophils were the first leukocytes to reach the airways in response to OVA nebulization during acute Th2 asthma model, we hypothesized that CXCR1 and CXCR2 could have an important role in orchestrating the subsequent steps of airway allergic inflammation. Then, we treated immunized mice with Ladarixin (18), a selective dual CXCR1 and CXCR2 non-competitive allosteric antagonist, 1 h before OVA nebulization and compared with the efficacy of dexamethasone, as depicted in protocol schedule **(** **Figure 3A** **)**. Ladarixin and dexamethasone treatment prevented the early macrophages and neutrophils accumulation into the airways 2 h after OVA nebulization, without changing eosinophil or lymphocyte influx as well as protein leakage into airways **(** **Figure 3B** **)**. OVA nebulization augmented CXCL1, CCL2, and CCL11 levels in lungs, but CCL2 and CCL11 were reduced after Ladarixin or dexamethasone treatment, whereas CXCL1 was reduced only by dexamethasone **(** **Figure 3C** **)**. Ladarixin also prevented neutrophil and eosinophil accumulation in lungs as depicted by MPO and EPO activities, similarly to dexamethasone **(** **Figure 3C** **)**. Given alone, Ladarixin and dexamethasone were able to improve the pulmonary mechanics in early moments by preserving lung volumes TLC, FRC, RV, and FEV100 **(** **Figure 3D** **)**, preserving the compliance and lung resistance, and most important, preventing bronchial hyperreactivity induced by methacholine **(** **Figure 3E** **),** as well as by improving the Flow-Volume curve **(** **Figure 3F** **)**. These results show that Ladarixin significantly blocks the acute manifestations of Th2 allergic airway inflammation and these effects were similar to those attained with dexamethasone treatment. Interestingly, CXCR1 and CXCR2 seem to contribute to neutrophil influx and airway hyperreactivity evoked by methacholine *in vivo*.

### Ladarixin Reduced Allergic Airway Inflammation in a Model of Repeated OVA Exposure

**Figure 4:**
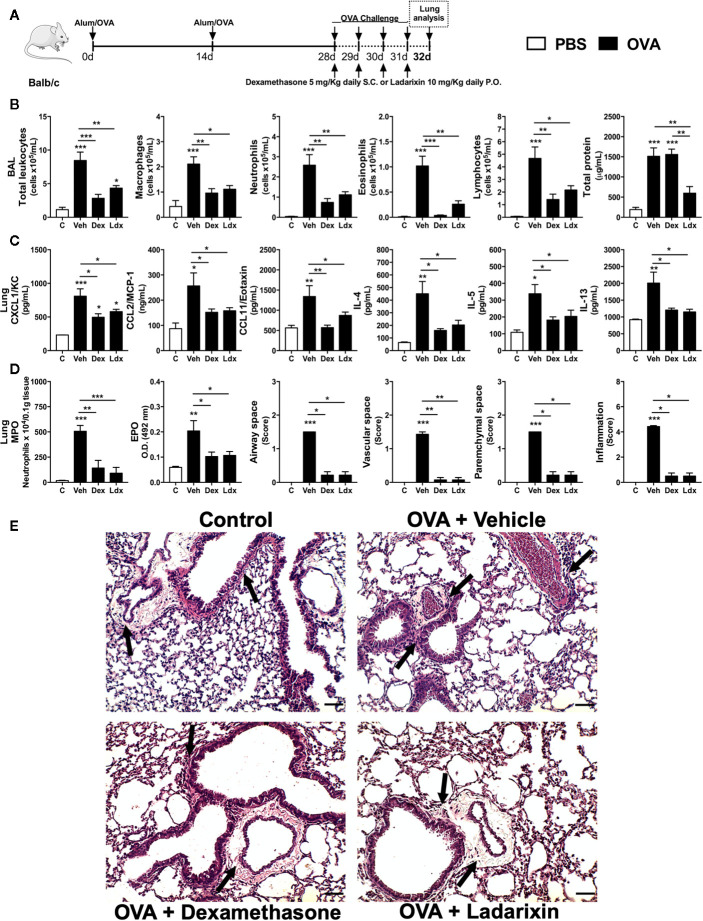
Ladarixin treatment reduces Th2 allergic airway and lung inflammation in acute asthma model. Inflammatory parameters were evaluated after four daily challenges with OVA. **(A)** Schematic representation of the experimental design. **(B)** Assessment of leukocyte influx and blood leakage into airways. **(C)** Chemokine and cytokine levels in lung parenchyma. **(D)** MPO and EPO activity quantification and pulmonary inflammation score. **(E)** Histological sections of lung (H&E dye). Arrows indicates peribronchial inflammation. n = 8 for each group, * for P < 0.05; ** for P < 0.01 and *** for p < 0.001.

We then performed a “classical” acute asthma model consisting of daily OVA nebulization carried out during four consecutive days. Under this condition, mice were treated with Vehicle, Dexamethasone or Ladarixin 1 h before each OVA airway exposures, as depicted in protocol schedule **(** **Figure 4A** **)**. Using this acute Th2 model we noted increased leukocyte numbers, characterized by increased eosinophil, neutrophil, macrophage and lymphocyte counts, accompanied by protein leakage into airways **(** **Figure 4B** **)**. In addition, there was increased lung production of the main Th2 cytokines IL-4, IL-5, and IL-13 and chemokines after OVA challenge **(** **Figure 4C** **)**. In line with the abovementioned results (**Figure 3** **)**, Ladarixin or Dexamethasone led to significant reduction in leukocyte airway influx, mostly due to decreased numbers of macrophages, neutrophils, eosinophils and lymphocytes, as **(** **Figure 4B** **)**. On the contrary to Ladarixin, Dexamethasone failed to reduce protein leakage into the airways **(** **Figure 4B** **).** Moreover, the production of CXCL1, CCL2, and CCL11 chemokines and Th2 cytokines IL-4, IL-5 and IL-13 were reduced by the treatments with either Ladarixin or Dexamethasone **(** **Figure 4C** **)**. Both Ladarixin and Dexamethasone isolated treatments also prevented neutrophil and eosinophil accumulation in lung parenchyma as assessed, respectively, by MPO and EPO activities **(** **Figure 4D** **)**. In tissue sections stained with H&E, there was no inflammation in control mice. In vehicle given challenged mice, leukocytes accumulated predominantly in lung parenchyma, but also in airways, perivascular and peribronchiolar areas following OVA challenge, as indicated by the arrows **(** **Figures 4D, E** **)**. Ladarixin or Dexamethasone were able to reduce lung inflammation in immunized and challenged mice (arrows) **(** **Figures 4D, E** **)**, suggesting that both drugs were equally effective in controlling “classical” Th2 allergic airway inflammation.

### Ladarixin Reduced Allergic Airway Inflammation, Remodeling, and Bronchial Hyperreactivity in a Model of Chronic OVA Exposure

**Figure 5:**
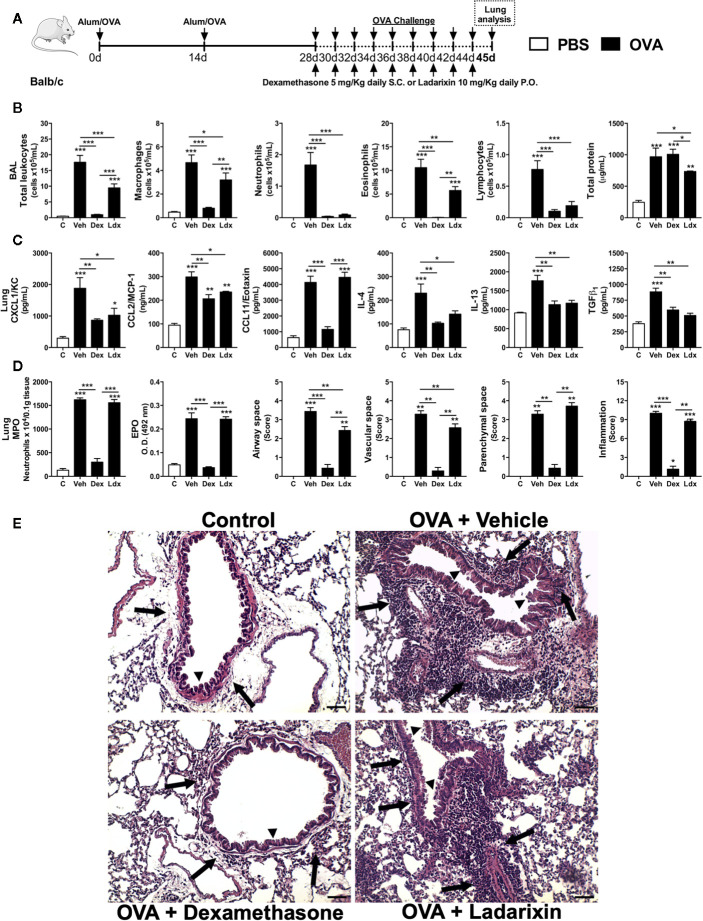
Ladarixin reduces Th2 allergic airway and lung inflammation in chronic asthma model. Inflammatory parameters were evaluated after 8 OVA challenges given every other day. **(A)** Schematic representation of the experimental design. **(B)** Assessment of leukocyte influx and blood leakage into airways. **(C)** Chemokine and cytokine levels in lung parenchyma. **(D)** MPO and EPO activity quantification and pulmonary inflammation score. **(E)** Histological sections of lung (H&E dye). Arrows indicates peribronchial inflammation. Arrows head indicates mucus production. n = 8 for each group, * for P < 0.05; ** for P < 0.01 and *** for p < 0.001.

To investigate if Ladarixin could be protective in the context of chronic airway inflammation and pulmonary remodeling observed in asthma, we used a second model of chronic asthma. In this model, mice were nebulized with OVA in alternated days during 16 days and treated daily with Vehicle, Dexamethasone or Ladarixin, as depicted in protocol schedule **(** **Figure 5A** **)**. Challenged mice presented elevated serum levels of IgA, IgG and IgE antibodies against OVA, which remained unaltered in mice subjected to Ladarixin treatment **(** **Supplementary Figure 1** **)**. In this chronic Th2 model, Ladarixin was less efficacious than Dexamethasone, but it still reduced total leukocytes infiltration in alveolar space as well as macrophages and eosinophils, with more pronounced blockade in infiltration of neutrophils and lymphocytes, and slight reduction in protein leakage into the airways **(** **Figure 5B** **)**. Dexamethasone failed to reduce protein leakage into the airways **(** **Figure 5B** **)**. Ladarixin was also able to reduce the CXCL1 and CCL2 but not CCL11 levels in a similar way to Dexamethasone **(** **Figure 5C** **)**. The Th2 cytokines and TGF-β1 levels were also marked decreased by Ladarixin or Dexamethasone treatment **(** **Figure 5C** **)**. Chronic OVA exposure caused a huge leukocyte accumulation in lung parenchyma dominated by neutrophils and eosinophils, that was prevented by Dexamethasone but not by Ladarixin **(** **Figure 5D** **)**. When we analyzed the lung histology, we observed that chronic exposure to OVA leads to perivascular and peribronchial inflammation (arrows) that was slightly reduced by Ladarixin to a lesser extent than Dexamethasone, without changing the inflammation in parenchymal space or total score of inflammation as depicted in **Figures 5D, E**.

**Figure 6:**
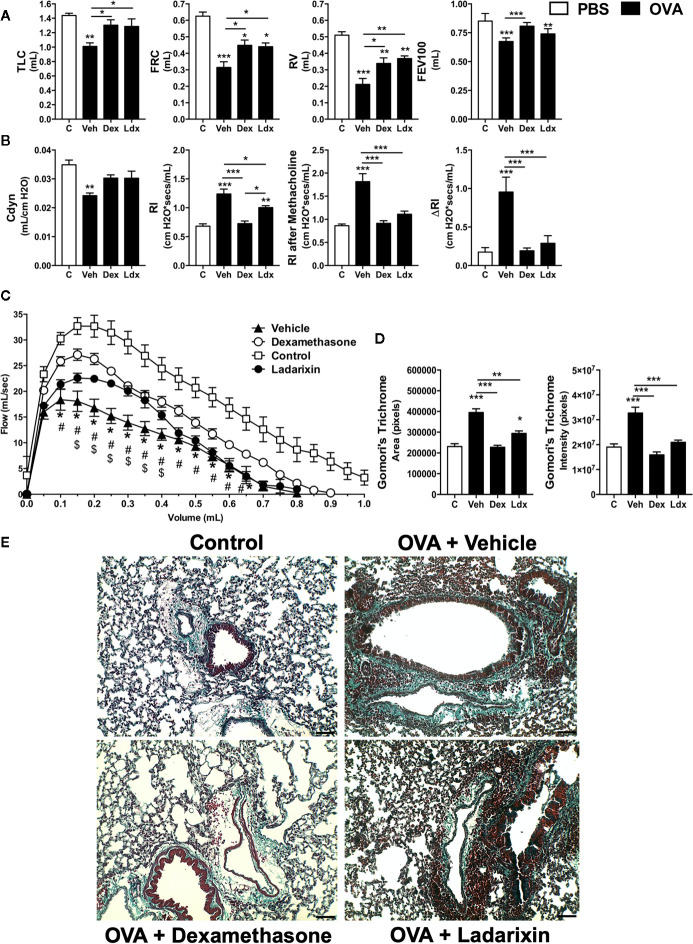
Ladarixin treatment decreases fibrosis and improves pulmonary function in chronic asthma model. Inflammatory parameters were evaluated after 8 OVA challenges given every other day. Lung fibrosis, as assessed by Gomori’s trichrome staining, and functional parameters were evaluated 24 h after the last challenge. Mice were treated with Ladarixin (10 mg/kg, gavage) or dexamethasone (5 mg/kg, s.c.) as indicated in . Pulmonary mechanics were assessed for **(A)** lung volumes, **(B)** compliance and lung resistance. **(C)** Flow-Volume curve. **(D)** fibrosis score. **(E)** Histological sections of lungs (Gomori’s trichrome dye). n = 8 for each group, * for P < 0.05; ** for P < 0.01 and *** for p < 0.001. TLC, total lung capacity; RV, residual volume; FRC, functional residual capacity; FEV100, forced expiratory volume in 100 ms; Cdyn, dynamic compliance; Rl, lung resistance; ΔRl, methacholine-evoked hyperresponsiveness. ^#^ differences between Dexamethasone vs Vehicle. ^$^ differences between Ladarixin vs Vehicle.

Chronic exposure to OVA by nebulization leads to pulmonary dysfunctions such as loss of volume, flow and elasticity. Ladarixin and Dexamethasone improved pulmonary mechanics during chronic Th2 airway inflammation, by reducing volume lost, such as TLC, FRC, RV, and FEV100 **(** **Figure 6A** **)**, preserving Compliance **(** **Figure 6B** **)**, reducing lung resistance, and most important, preventing bronchial hyperreactivity evoked by methacholine **(** **Figure 6B** **),** as well as improving airway flow, as depicted by the Flow-Volume curve **(** **Figure 6C** **)**.

Chronic OVA nebulization also provoked tissue remodeling marked by collagen deposition in peribronchial regions of lungs, as shown in Gomori’s Trichrome stained lung slides **(** **Figures 6D, E** **)**. Dexamethasone and Ladarixin reduced collagen deposition in peribronchial areas of lung parenchyma when compared to Vehicle, as evaluated by Gomori’s trichrome morphometry **(** **Figure 6D** **)** and depicted in **Figure 6E** photomicrography. We analyzed lung sections stained with periodic acid-Schiff (PAS) to assess goblet cell metaplasia and mucus production related to allergic airway inflammation **(** **Supplementary Figure 2** **)**. Vehicle mice showed an increased goblet cell metaplasia and mucus production (arrowheads) **(** **Supplementary Figures 2A, B** **)**, which were prevented by Dexamethasone **(** **Supplementary Figures 2A, B** **)**. However, Ladarixin failed to prevent the mucus overproduction and goblet cell hyperplasia (arrowheads) after chronic OVA exposure **(** **Supplementary Figures 2A, B** **)**.

### Ladarixin Reduced Pulmonary Inflammation and Fibrosis Induced by Bleomycin in Mice

**Figure 7:**
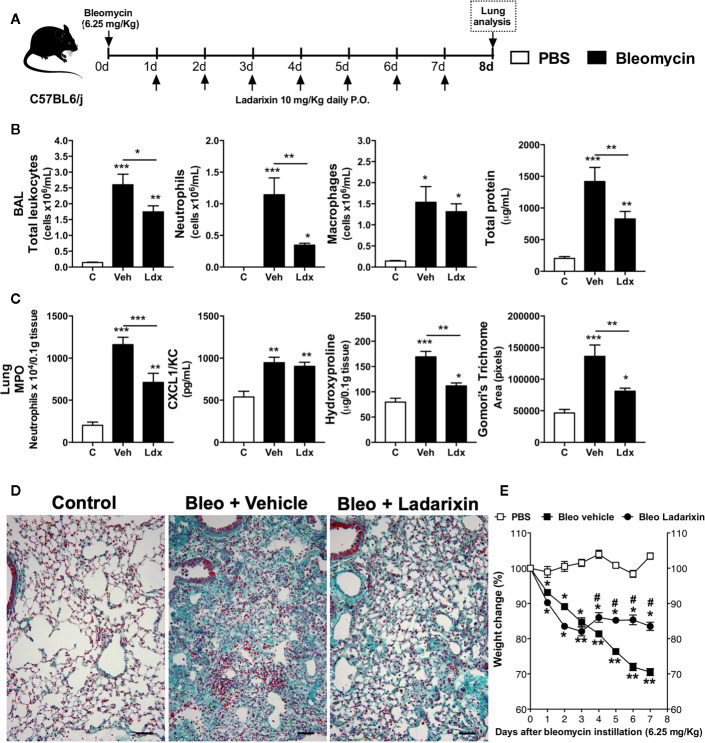
Ladarixin treatment decreases fibrosis and airway inflammation in a Bleomycin-induced lung fibrosis model. **(A)** Schematic representation of the experimental design. **(B)** Assessment of leukocytes influx and blood leakage into airways. **(C)** MPO activity quantification, CXCL1/KC levels and fibrosis assessment. **(D)** Histological sections of lungs (Gomori’s trichrome dye). **(E)** Mice weight change after Bleomycin instillation. n = 8 for each group, * for P < 0.05; ** for P < 0.01 and *** for p < 0.001. ^#^ differences between Ladarixin vs Vehicle.

Based on observations that Ladarixin reduces airway remodeling in a model of chronic asthma **(** **Figures 6D, E** **)**, we further investigated whether Ladarixin could also reduce lung fibrosis induced by bleomycin in mice. This model is frequently used to evaluate molecules in context of neutrophilic inflammation and fibrosis induced by an epithelial injury and excessive fibroblast activation and tissue scaring (20). We used a high dose of bleomycin instillation (6.25 mg/kg) and after 8 days we performed the analyses, as depicted in protocol schedule **(** **Figure 7A** **)**. Bleomycin instillation led to increased inflammation, marked by mixed neutrophil and macrophage influx and protein leakage into airways in Vehicle mice **(** **Figure 7B** **)**. Bleomycin also induced increase in MPO activity and CXCL1 levels in lungs **(** **Figure 7C** **)** as well as collagen accumulation in parenchyma **(** **Figures 7C, D** **)** in which reflects in mice weight loss **(** **Figure 7E** **)**. However, Ladarixin administration reduced airway inflammation, protein leakage **(** **Figure 7B** **)** and neutrophil accumulation in lungs as assayed by MPO **(** **Figure 7C** **)**. Ladarixin also reduced lung fibrosis as assessed by hydroxyproline assay and Gomori’s trichrome staining and morphometry **(** **Figures 7C, D** **)**. Ladarixin also attenuated weight loss in animals challenged with bleomycin **(** **Figure 7E** **)**. These results show that Ladarixin may have a key role in controlling neutrophilic airway inflammation and tissue fibrosis, similarly to what was observed during a chronic Th2 allergic airway inflammation.

### Ladarixin Reduced Airway Inflammation, Remodeling, and Bronchial Hyperreactivity in a Model of Th17-Dependent Steroid-Insensitive Asthma

**Figure 8:**
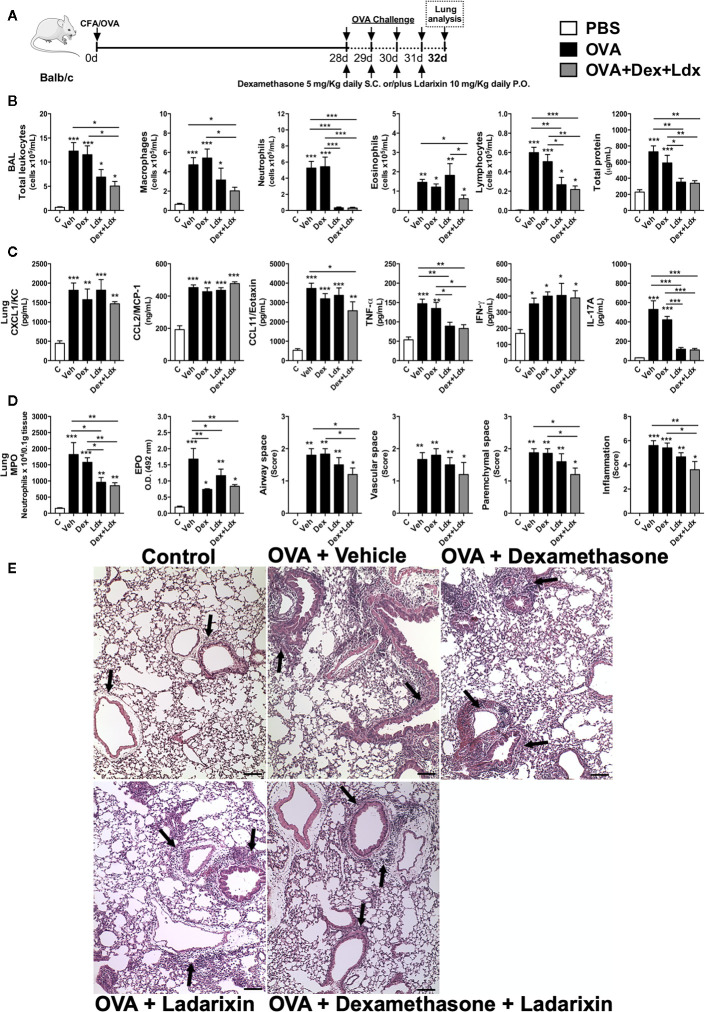
Ladarixin treatment decreases pulmonary inflammation in an acute Th1/Th17 glucocorticoid-refractory neutrophilic asthma model. Inflammatory parameters were evaluated after four daily challenges with OVA. **(A)** Schematic representation of the experimental design. **(B)** Assessment of leukocyte influx and blood leakage into airways. **(C)** Chemokine and cytokine levels in lung parenchyma. **(D)** MPO and EPO activity quantification and pulmonary inflammation score. **(E)** Histological sections of lung (H&E dye). Arrows indicate peribronchial inflammation. n = 8 for each group, * for P < 0.05; ** for P < 0.01 and *** for p < 0.001.

Patients with severe asthma present a predominance of neutrophils in the airways and are refractory to steroids/corticoids. Further experiments were designed to investigate the effects of Ladarixin in a model of Th17-dependent model of allergy that is mostly neutrophilic and not ameliorated by treatment with steroids (27, 30). In this model, mice were immunized with OVA in CFA at day 0 and after 28 days mice were nebulized with OVA during four consecutive days, as depicted in protocol schedule **(** **Figure 8A** **)**. We found a significant leukocyte influx into the airways of Vehicle treated mice with predominance of neutrophils and macrophages, and less extended numbers of eosinophils and lymphocytes, accompanied by increased protein leakage **(** **Figure 8B** **)** in the context of the classical Th17 cytokines *milieu* in lungs (**Figure 8C** **)**. While the treatments with CXCR1 and CXCR2 antagonist alone or in combination with Dexamethasone were able to reduce leukocyte influx into alveolar space, by marked reduction in numbers of macrophages and neutrophils, Dexamethasone failed to reduce the recruitment of these cells **(** **Figure 8B** **)**. Although Ladarixin alone did not prevent eosinophil recruitment into the airways, co-treatment with Dexamethasone plus Ladarixin reduced the recruitment of these cells. Unlike the use of Dexamethasone alone, Ladarixin *per se* or combined with Dexamethasone administration was able to reduce lymphocyte influx and protein leakage into the airways **(** **Figure 8B** **)**. Moreover, levels of CXCL1 and CCL2 chemokines were not affected by Ladarixin, Dexamethasone, or Ladarixin plus Dexamethasone, whereas CCL11 levels were reduced by the combined treatment **(** **Figure 8C** **)**. The Th17 cytokines IL-17A and TNF-α were also markedly decreased by Ladarixin alone or in combination with Dexamethasone, but not with Dexamethasone alone **(** **Figure 8C** **)**. Lung levels of IFN-γ were not affected by any treatment **(** **Figure 8C** **)**.

**Figure 9:**
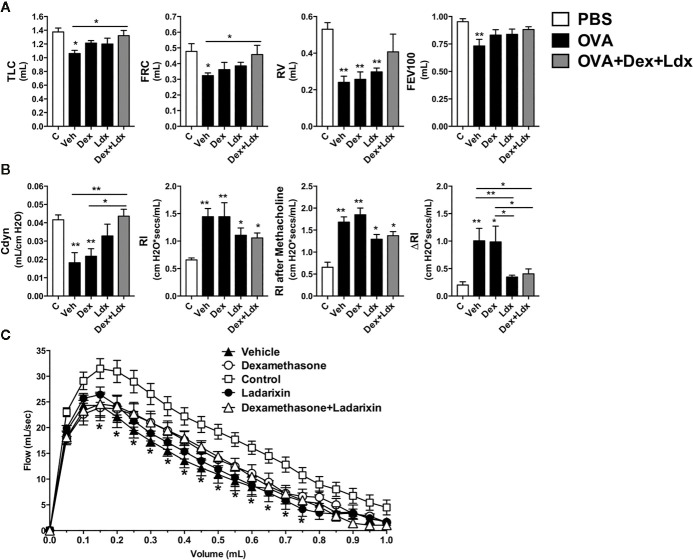
Ladarixin treatment ameliorates pulmonary function in a Th1/Th17 GC-refractory neutrophilic asthma model. Inflammatory parameters were evaluated after four daily challenges with OVA. **(A)** Lung volumes. **(B)** compliance and lung resistance. **(C)** Flow-volume curve. n = 8 for each group, * for P < 0.05 and ** for P < 0.01. TLC, total lung capacity; RV, residual volume; FRC, functional residual capacity; FEV100, forced expiratory volume in 100 ms; Cdyn, dynamic compliance; Rl, lung resistance; ΔRl, methacholine-evoked hyperresponsiveness.

When we evaluated the leukocyte infiltration into lung tissue, Ladarixin and Dexamethasone plus Ladarixin treatments were able to prevent the neutrophil and eosinophil accumulation in lung parenchyma after OVA exposure, as assessed by MPO and EPO activities **(** **Figure 8D** **)**. By evaluating the lung histology and pathology score, we could observe that there is no inflammation in control mice, but in OVA immunized mice treated with Vehicle the leukocytes accumulate predominantly in lung parenchyma, but also in airway, perivascular and peribronchial areas **(** **Figure 8D** **)**, as indicated by arrows **(** **Figure 8E** **)**. Only the combined treatment with Ladarixin plus Dexamethasone reduced inflammation as measured by score of lungs from immunized mice (arrows) after OVA challenge **(** **Figures 8D, E** **)**. Th17 airway inflammation leads to tissue dysfunction such as loss of lung volumes, reduced airway flow and pulmonary elasticity that was insensible to Dexamethasone treatment **(** **Figure 9** **)**. However, Ladarixin and Dexamethasone combined treatment improved the lung volumes TLC and FRC **(** **Figure 9A** **),** and with preserved Compliance **(** **Figure 9B** **)**. Most important, Ladarixin as well as Dexamethasone plus Ladarixin were able to reduce airway hyperreactivity following methacholine **(** **Figure 9B** **)**, but no changes in the airway flow were observed, as depicted by the Flow-Volume curve **(** **Figure 9C** **)**. Collectively these results show that Ladarixin has anti-inflammatory effects during corticosteroid-insensitive Th17 neutrophilic airway inflammation and hyperreactivity induced by OVA nebulization in mice.

### Ladarixin Protects Mice From Cigarette Smoke-Induced Exacerbation of Influenza-A Infection

**Figure 10:**
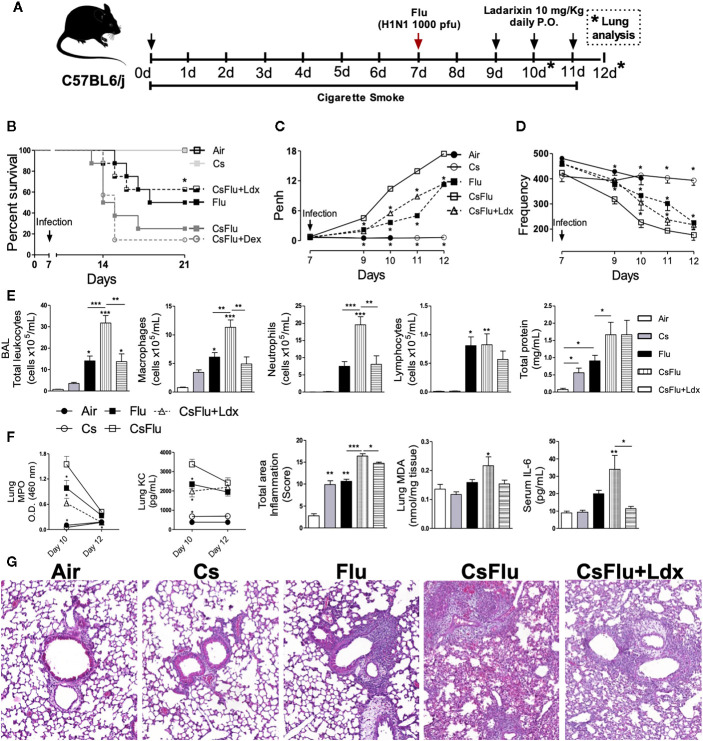
Ladarixin protects mice from cigarette smoke-induced exacerbation of Influenza-A infection. **(A)** Schematic representation of the experimental design. **(B)** Survival proportions of the different groups of mice after day 7 (infection day). **(C)** Effect of Ladarixin on cigarette smoke (Cs)-induced exacerbation of Penh elevation in mice subjected to H1N1 infection. **(D)** Effect of Ladarixin over respiratory frequency on mice subjected to H1N1 infection and cigarette smoke (CsFlu). **(E)** BALF analysis at day 12. Assessment of leukocyte influx and protein leakage into airways **(F)** (From right to left) Comparison of MPO activity and KC levels in lung tissue at days 10 and 12; inflammation score, derived from histological analyses, from lung tissue obtained at day 12; MDA levels in lung tissue at day 12; IL-6 levels in mice serum at day 12. **(G)** Representative histological sections of lung (H&E dye) for each group obtained at assay day 12. n≥6 for each group * for P < 0.05; ** for P < 0.01 and *** for p < 0.001. For **(B–D)** * represents significant differences compared to CsFlu group. In MPO and KC graphs, * represents significant differences compared to CsFlu group. For the rest of the graphs in (E, F)* represents significant differences compared to Air group except when indicated with a solid line.

We further investigated the effect of CXCR1/2 antagonism by Ladarixin on cigarette smoke (Cs)-induced exacerbation of mortality and lung pathological changes induced by influenza A infection in mice as depicted in **Figure 10A**. Viral infection alone (Flu group) led to 50% mice lethality evaluated 2 weeks post-infection. The combination of Cs exposure and viral infection (CsFlu group) provoked a faster and increased lethality (85%) while no lethality was detected in Air and CS groups as expected. It is noteworthy that CsFlu group exacerbation of mortality was resistant to oral treatment with dexamethasone (1 mg/kg) at days 9, 10 and **(** **Figure 10B** **).** In another experimental setting, we found significant alterations of lung function in virus-infected mice (Flu) 2 to 5 days post-infection, characterized by elevation in Penh and decrease in respiratory frequency values, which were clearly exacerbated when infected mice were exposed to CS (CSFlu mice) **(** **Figures 10C, D**, respectively).7 The same figures show no significant changes in non-infected mice exposed to either ambient air or Cs. Ladarixin (10 mg/kg) orally given at days 2, 3 and 4 post-infection significantly attenuated the exacerbation in lethality **(** **Figure 10B** **)** and respiratory changes **(** **Figures 10D, C** **)** noted in CSFlu group.

Numbers of total leukocytes in the BAL fluid appeared 5-, 20-, and 45-fold increase in Cs, Flu, and CsFlu mice, respectively, compared to those exposed to ambient air on day 12 **(** **Figure 10E** **)**. As shown in **Figure 10E**, macrophages accounted for by the majority of leukocytes in Cs mice, while a mix of macrophages, neutrophils and lymphocytes accounted for by leukocyte number elevations in Flu mice. Leukocyte exacerbated numbers observed in BAL of CsFlu mice was underlined by 85% increase in macrophage and 160% increase in neutrophil numbers as compared to non-smoking Flu mice. All changes but lymphocyte numbers were inhibited by Ladaraxin **(** **Figure 10E** **)**. Protein leakage in the airways was similarly increased by Cs exposure or Flu infection, nevertheless, the combination of both insults (CsFlu) exacerbated protein leakage that remained unaltered in Ladaraxin treated mice **(** **Figure 10E** **)**. In lung tissue, we observed an exacerbated neutrophil presence in CsFlu mice compared to Flu mice, measured by MPO activity at day 10. Instead, MPO activity was comparable between CsFlu and Flu groups at day 12. We detected no significant MPO activity in Cs or Air mice at day 10 or 12. In addition, KC levels in lung tissue at day 10 were also exacerbated in CsFlu mice compared to Flu mice **(** **Figure 10F** **)**. As assessed by histological analysis, total inflamed area in CsFlu mice appeared augmented as compared to those noted in CS and Flu mice at days 10 and 12 **(** **Supplementary Figures 3A, D** **;** **Figures 10F, G** **)**. Furthermore, lipid peroxidation of lung tissue, assessed by MDA quantification, was significantly increased only in the CSFlu group **(** **Figure 10F** **)**. Viral load in lung tissue was comparable in flu and Csflu groups, both showing equally reduced levels of IL-10 as compared to Cs, which remained unaltered in comparison to the Air group **(** **Supplementary Figures 3B, C** **)**. Regarding systemic inflammatory changes, Flu infected mice increased serum IL-6 levels, which were exacerbated in CsFlu mice **(** **Figure 10F** **)**.

Ladarixin treatment reduced lung MPO activity and KC levels of CsFlu mice at day 10 as well as lung tissue inflammation of CsFlu mice at days 10 and 12 **(** **Supplementary Figures 3A, D** **;** **Figures 10F, G** **)**. Consequently, Ladarixin treatment inhibited the exacerbated accumulation of leucocytes in BALF of CsFlu mice at day 12 with a remarkable impact over neutrophil infiltration. It also prevented lipid peroxidation in lung tissue and restored IL-10 levels to control values. Finally, exacerbation of serum IL-6 levels was avoided by Ladarixin **(** **Figures 10E, F** **)**, but no changes in viral replication were found **(** **Supplementary Figure 3B** **)**. Briefly, the blockade of CXCR1/2 protects mice from lethality and lung function impairment in a model of AECOPD triggered by influenza A infection by reducing neutrophil infiltration and preventing oxidative stress and systemic inflammation.

## Discussion

The increasing impact of respiratory disorders such as asthma, fibrosis and COPD in human health brings up the need of novel and effective therapeutics. Despite their heterogeneous onset and pathogenesis, these disorders are frequently associated with chronic neutrophilic inflammation and impairment of lung function. Ladarixin is a dual CXCR1 and CXCR2 non-competitive allosteric antagonist that presents a long half-life and oral bioavailability (18). It interacts with a conserved site in CXCR1 and CXCR2, preventing chemokine-induced receptor activation and neutrophil chemotaxis, and reducing the inflammatory response (18). For that reason, phase 2 clinical trials are being conducted to study the therapeutic potential of Ladarixin in the prevention of pancreatic β-cell loss as a consequence of dysregulated neutrophilic activity, thus delaying disease progression, in patients at the onset of type 1 diabetes (NCT02814838). In this context, here we demonstrated that Ladarixin could represent a new therapeutic strategy to treat a wide range of acute and chronic airway disorders with a neutrophilic component, as depicted in the **Graphical Abstract**. Therefore, along this work we presented strong pre-clinical data showing that Ladarixin prevented inflammation, lung damage and pulmonary dysfunction in i) acute or chronic onset classic Th2-eosinophilic asthma; ii) in a model of Th17-neutrophilic and GC-insensitive asthma models; iii) in a model of pulmonary inflammation and fibrosis induced by bleomycin; and in iv) a model of acute inflammatory exacerbation of influenza infection triggered by cigarette-smoke exposure.

More than 50 cytokines and chemokines, secreted by resident lung parenchyma cells and resident or infiltrating leukocytes, have been described to play a key role in the development and maintenance of asthma (37, 38). In spite of being a heterogeneous disorder, asthma is classically considered as a Th2 (39) and eosinophilic dominant disorder (26), which can be successfully controlled with inhaled glucocorticoids (6, 40). However, 5-10% of asthma patients present the severe form of the disease and are refractory to glucocorticoids (6, 40). In these cases, the inflammatory profile is changed to a mixed Th17 milieu with increased neutrophil influx into the airways (41). In this work we used a classic model of Th2 eosinophilic airway inflammation, and, an alternative model of Th17 neutrophilic airway inflammation in which mimics to GC-refractory asthma to better understand the role of CXCR1 and CXCR2 receptors in different manifestations of asthma. Using a classic Th2 asthma model, we found that neutrophils are the first cells to be recruited into the airways. Moreover, in the experiments performed with ΔdblGATA-1 animals that lack eosinophils (26), mice developed bronchial hyperreactivity against methacholine given at 2 h after a single OVA challenge, despite the absence of significant leukocyte infiltration. At that time point increased tissue levels of CXCL1, the murine analogous of human CXCL8. CXCL8 may also have direct bronchoconstrictor effects on human airway smooth muscle cells (42), which express CXCR1 and CXCR2. Inhibition of CXCR1 and CXCR2 by Ladarixin prevented airway hyperreactivity, the main asthma manifestation in those animals. Thus, this effect may also be explained by the previously demonstrated ability of CXCL8 to induce human airway smooth muscle contraction (42). Treatment with Ladarixin, similarly with Dexamethasone, reduced Th2 allergic airway and lungs inflammation. Contrary to Ladarixin, however, Dexamethasone failed to reduce protein leakage into airways. CXCR2 is involved in a hypersecretory response in lungs and (43), CXCR2 blockage may explain this reduced protein levels in BALF from Ladarixin compared to Dexamethasone treated mice.

In people with mild to moderate asthma, there is predominant accumulation of eosinophils and CD4+ cells that produce Th2 cytokines (39) in the lungs and high concentration of serum IgE. In patients with severe asthma, there appears to be predominance of neutrophils in the airways (44) with less reversible airway obstruction and a Th17 cytokine milieu (41, 45, 46). The limited success of GCs in these severe patients has added support to the interpretation that asthma is a heterogeneous disease and that we may improve therapy by segregating asthmatic patients based on their inflammatory environment and their response to GCs (38, 40, 47). Mice treated with Ladarixin in Th2 or Th17-skewed airway inflammation models presented low levels of the classic Th2 (IL-4, IL-5, and IL-13) and Th17 (IL-17A and TNF-α) cytokines, respectively. Of importance, Ladarixin per se or in combination with GC were effective in GC-refractory asthma. This was accompanied by reduced levels of CXCL1, CCL11, and CCL2 chemokines and the consequent reduced inflammation in lungs and airways which contributed to the improvement of pulmonary mechanics, the main cause of asthma complications and worsening of life quality of patients with asthma. In this sense, Ladarixin treatment reduced not only eosinophils, neutrophils and lymphocytes migration to the airways, but also reduced the number of macrophages. The reduction in macrophages can be due to the reduction in the CCL2 chemokine production in the airway, but also, it could be due to Ladarixin acting directly in CXCR2 expressed in monocytes and in macrophages. The presence of IL-13 and IL-4 has been shown to up-regulate the expression of CXCR1 and CXCR2 in both human-derived monocytes and M2 macrophages. The increased expression of these receptors resulted in an acquired responsiveness of monocytes to CXCL8, inducing directional migration and activation of respiratory burst in monocytes (48). In our chronic Th2 asthma model, we found that CXCR1 and CXCR2 has an important role in airway remodeling since Ladarixin treatment reduced peribronchial collagen deposition and the low levels of, well-known, pro-fibrogenic cytokines IL-4, IL-13, and TGF-β1 (15). Regarding the role of CXCR1 and CXCR2 in the context of fibrosis, the increased CXCL8 levels found in airways from IPF patients were associated with neutrophilic infiltration and disease severity (16, 17). Indeed, we have previously evaluated the role of CXCR1 and CXCR2 in the context of experimental pulmonary fibrosis (20). The blockade of CXCR1 and CXCR2 with a Ladarixin analogue, DF2162, clearly prevented the neutrophilic influx but also angiogenesis and fibrosis by acting on endothelial cells and fibroblasts (18, 20), leading to attenuated TGF-β1 levels, reduced angiogenesis and collagen deposition. Collectively, Ladarixin displayed anti-fibrogenic effects in both models of chronic Th2 allergic airway inflammation and bleomycin-induced pulmonary fibrosis.

The anti-inflammatory performance of Ladarixin in AECOPD model was also very promising since a short-term therapeutic treatment could control the inflammatory exacerbation and also prevented the increasing mice mortality when corticosteroid treatment could not. In this sense, Ladarixin treatment prevented leukocyte influx exacerbation towards the airways of CsFlu mice, mostly by reduction of neutrophil numbers but also affecting macrophage accumulation. It is very likely that Ladarixin’s effect over the airways resulted from the reduction in neutrophil activity and KC levels observed in lung tissue earlier than airways in treated CsFlu mice. Histological evaluation of CsFlu mice lungs also showed a positive anti-inflammatory effect of Ladarixin treatment. Elevated recruitment of neutrophils to the airways has been extensively associated with impairment of lung function and increase of oxidative species (49). In agreement, the reduction in neutrophil influx into the airways and consequent reduction of oxidative stress following Ladarixin treatment led to an improvement of lung function in CsFlu mice. Importantly, neither the accentuated physiological deterioration of CsFlu group nor its improvement by the treatment is a consequence of differential viral replication since all groups showed comparable viral titer. Nonetheless, and corroborating our data from models of allergic Th2 and Th17 airway inflammation, we cannot discard the possible effects of Ladarixin acting directly over CXCR1 and CXCR2 from peribronchial smooth muscle cells (42) in AECOPD model. Systemic inflammation is well described in COPD patients and augmented IL-6 serum levels have been associated with higher occurrence of exacerbations and poor prognosis (50). Interestingly, the combination of Cs and viral infection in our model augmented the levels of IL-6 in serum which was inhibited by Ladarixin, protecting mice from a severe systemic inflammatory condition. Since neutrophils are a very important source of IL-6 (51), Ladarixin administration may protect mice from systemic inflammation by reducing neutrophil activity in our model. Thus, by controlling neutrophils activity and reducing lung tissue damage Ladarixin increased mice survival.

Ladarixin is a negative allosteric modulator of CXCR1 and CXCR2 chemokine receptors, which means that it acts either by stabilizing an inactive conformation of the receptor or by raising the energy barrier necessary to activate the receptor. Ladarixin, however does not affects the cognate ligand binding affinity, in other words, Ladarixin blocks the signal transduction of CXCR1 and CXCR2 without altering the binding site of natural ligands, making these receptors now acts as scavenger receptors for the endogenous agonists (18). The CXCR2 chemokine receptor is found both on immune and structural cells in the lungs (3). Thus, these promising results of Ladarixin in the treatment of a wide range of acute and chronic airway disorders should be, not only due to its effects on neutrophils, but also due to its effects on other cells. Therefore, Ladarixin will block CXCR2 in a wide range of cells, including immune and lung structural cells. In this sense, CXCR2 is expressed on endothelial cells in the lungs and may be linked to the augmented angiogenesis in the airway mucosa, a characteristic of both human asthma and COPD (52). It has been shown that CXCR2 expression on endothelial cells has a key feature in the antigen-induced recruitment of mast cells into the lungs by regulating VCAM-1 expression (53). There are evidences showing that CXCR2 may be involved in mucus overproduction in lungs (54), another possible target of Ladarixin. CXCR2 is also expressed on airway smooth muscle cells (55, 56) and in this work we showed that CXCR2 may be a key feature in the development of airway hyperresponsiveness and, Ladarixin may be acting on CXCR2 expressed on ASM cells to improve the airways hyperresponsiveness. Although CXCR2 was not found to be expressed in fibroblasts isolated from healthy patients, it is expressed on fibroblasts during fibroproliferative diseases (20, 57). In this sense, the reduced fibrosis induced by Ladarixin treatment, in both chronic asthma and Bleomycin-induced fibrosis model, may be, also, due to the blockage of CXCR2 on fibroblasts.

Several clinical trials targeting CXCR2 or ELR+ chemokines in pulmonary disorders are currently underway (1). Navarixin, a selective CXCR1 and CXCR2 antagonist, was tested in patients with allergen-induced asthma and severe neutrophilic asthma. Although the treatment reduced sputum myeloperoxidase, IL-8 levels, elastase and neutrophils numbers (58), no apparent improvement of respiratory parameters in treated patients was reported (NCT00688467 and NCT00632502). Recently, a small clinical trial involving patients with acute influenza infection was conducted using Danirixin (59), where the authors concluded that the treatment was well tolerated and did not impede influenza viral clearance (NCT02130193). COPD patients treated with selective CXCR2 receptor antagonists, Navarixin or Danirixin, presented a significant improvement in FEV1 compared to placebo group and suggested a clinically important anti-inflammatory effect of the CXCR2 antagonism (60). Even though CXCR1/2 inhibition could impair other important functions of neutrophils increasing host vulnerability to pathogen infections, animal models and clinical trials have shown that the treatment is safe (61). It is estimated that a healthy human produce daily about 1 x 106 neutrophils per kilogram. It has been demonstrated that CXCR2 expression is important in neutrophil egress from bone marrow (62). In this sense, chronic antagonism of CXCR2 chemokine receptor could impair neutrophil trafficking from bone marrow and cause neutropenia. Although, greater treatment response was observed in smokers versus ex-smokers (NCT01006616). Even though no difference was observed in the number of HCRU-defined exacerbations experienced by participants in both groups, the data raise the possibility that Danirixin may reduce the duration of COPD exacerbation (63) (NCT02130193). We must highlight that inflammatory exacerbation in COPD patients is responsible for most hospitalizations, and enhances disease mortality, with no effective treatment to control them (64, 65). Given that we believe that Ladarixin could specifically control the exacerbation process and might bring substantial benefits not only for COPD patients, but also extended to asthma and flu infection. In this direction, Danarixin and Navarixin inhibited both CXCR1 and CXCR2 functions in most of the clinical trials of COPD, asthma and influenza infection.

Indeed, CXCR1 and CXCR2 antagonism through oral administration of Ladarixin prevented neutrophilic airway inflammation and protein leakage, peribronchial tissue remodeling, interstitial fibrosis and bronchial hyperreactivity improving pulmonary mechanics in all human disease-related models used here, including GC-sensitive and insensitive asthma, and enhanced mice survival in AECOPD model (**Graphical Abstract**). Although glucocorticoids are very effective anti-inflammatory drugs, severe asthmatics and COPD patients show poor response to steroids-based therapeutics. For that reason, this work explores two contexts. Steroid-sensitive models, where we used dexamethasone as a control drug to compare the effects achieved by administration of Ladarixin. And steroid-refractory models, such as Th17-dependent asthma (CFA+OVA) and AECOPD where we demonstrated that dexamethasone had no anti-inflammatory effect. In this sense, these two last models are of great importance for the study and characterization of drugs that act in refractory corticotherapy conditions, *per se* or in combination to the other drugs, to further evaluated the reversal of refractoriness and potentiation of effects. Here we showed that Ladarixin was effective in two pre-clinical GC-insensitive mice models, Th17 asthma and AECOPD. The lack of alternative treatments has a direct impact in those patient outcomes, reducing quality of life health and increasing the rate of symptoms exacerbations, number of hospitalizations and mortality, as well as burden on health care systems. Both, severe asthmatics and COPD patients, present strong neutrophilic activity in lung tissue that contribute significantly to inflammation-mediated tissue damage, deterioration of lung function and it has been often associated with glucocorticoids resistance development. In fact, phase 2 interventional clinical trials on asthma, influenza infection or COPD have been already performed using the CXCR1 and CXCR2 inhibitors. Therefore, Ladarixin emerges as an important drug candidate to treat a broad range of respiratory diseases and could potentially retrieve better outcomes in combination with steroids than the candidates already tested for the treatment of neutrophilic lung diseases.

## Data Availability Statement

The raw data supporting the conclusions of this article will be made available by the authors, without undue reservation.

## Ethics Statement

The animal study was reviewed and approved by Ethical committee CEUA/Federal University of Minas Gerais and Ethical committee CEUA/Oswaldo Cruz Institute.

## Author Contributions

CG, MM, MT, and RR designed research. MM, MF, LK, GL, DR, GC, FO, and CG performed experiments. MM, MF, LK, GL, DR, GC, FO, CG, and RR analyzed data. MM, MF, CG, MM, MT and RR and wrote the manuscript. LB and MA provided the compound and contributed with manuscript revision. All authors contributed to the article and approved the submitted version.

## Funding

This investigation received financial support from Fundação de Amparo à Pesquisa do Estado de Minas Gerais (FAPEMIG, Brazil) under Grant Agreement No. CBB-APQ-03570-16 Edital 01/2016—Demanda Universal/FAPEMIG and Grant Agreement No. CBB-PPM 00508-18 Edital 02/2018—Programa Pesquisador Mineiro/FAPEMIG; Produtividade em Pesquisa—PQ Conselho Nacional de Desenvolvimento Científico e Tecnológico (CNPq, Brazil) under Grant Agreement No. 309810/2017-5 Edital 12/2017, Fundação de Amparo à Pesquisa do Estado do Rio de Janeiro (FAPERJ, Brazil) under Young Scientist Grant JCNE E-26/203.156/2017 to CG; Comissão de Aperfeiçoamento de Pessoal de Ensino Superior (CAPES, Brazil). This project was also supported by the Instituto Nacional de Ciência e Tecnologia-INOFAR, Brazil (CNPq Grant Agreement No. 465249/2014-0), and by the European Union Seventh Framework Program (FP7-2007/2013, TIMER consortium) under Grant Agreement No. HEALTH-F4-2011-281608.

## Conflict of Interest

The authors LB and MA were employed by the company Dompé Farmaceutici S.p.a., L’Aquila, Italy.

The remaining authors declare that the research was conducted in the absence of any commercial or financial relationships that could be construed as a potential conflict of interest.

## Supplementary Material

Supplementary Figure 1 Mice atopy induced by OVA challenge. IgA, IgG and IgE antibodies levels were measured by ELISA. n = 8 for each group, * for P < 0.05; ** for P < 0.01.

Supplementary Figure 2 Ladarixin treatment did not reduces mucus overproduction. Mucus production was evaluated after four daily challenges with OVA. **(A)** Histogram showing area positive for mucus, which indicates goblet cell hypertrophy. **(B)** Lung histological sections (Periodic acid-schiff dye). Arrows head indicate goblet cells hypertrophy and mucus overproduction. n = 8 for each group, ** for P < 0.01.

Supplementary Figure 3 Ladarixin effect over lung histological sections, viral titer and IL-10 production in lung tissue. **(A)** Inflammation score, derived from histological analyses from lung tissue, obtained at day 10 of Cs exposure. **(B)** Lung viral titer from infected mice groups on day 12 of assay. **(C)** IL-10 levels in lung tissue of mice obtained on day 12 of assay. **(D)** Representative histological sections of lung (H&E dye) for each group obtained at day 10 of Cs exposure. n = 6 for each group, * represents significant differences compared to Air group except when indicated with a solid line * for P < 0.05 and ** for P < 0.01.

## References

[1] Russo RC Garcia CC Teixeira MM Amaral FA The CXCL8/IL-8 chemokine family and its receptors in inflammatory diseases. Expert Rev Clin Immunol (2014) 10:593–619. 10.1586/1744666X.2014.894886 24678812

[2] Casilli F Bianchini A Gloaguen I Biordi L Alesse E Festuccia C Inhibition of interleukin-8 (CXCL8/IL-8) responses by repertaxin, a new inhibitor of the chemokine receptors CXCR1 and CXCR2. Biochem Pharmacol (2005) 69:385–94. 10.1016/j.bcp.2004.10.007 15652230

[3] Chapman RW Phillips JE Hipkin RW Curran AK Lundell D Fine JS CXCR2 antagonists for the treatment of pulmonary disease. Pharmacol Ther (2009) 121:55–68. 10.1016/j.pharmthera.2008.10.005 19026683

[4] Fanta CH Asthma. N Engl J Med (2009) 360:1002–14. 10.1056/NEJMra0804579 19264689

[5] GINA, Global Strategy for Asthma Management and Prevention, Global Initiative for Asthma pp. GINA Report 2019, Global Strategy for Asthma Management and Prevention. (2019). Available at: https://ginasthma.org/.

[6] Holgate ST Polosa R The mechanisms, diagnosis, and management of severe asthma in adults. Lancet (2006) 368:780–93. 10.1016/S0140-6736(06)69288-X 16935689

[7] Chanez P Wenzel SE Anderson GP Anto JM Bel EH Boulet LP Severe asthma in adults: what are the important questions? J Allergy Clin Immunol (2007) 119:1337–48. 10.1016/j.jaci.2006.11.702 17416409

[8] Dockrell M Partridge MR Valovirta E The limitations of severe asthma: the results of a European survey. Allergy (2007) 62:134–41. 10.1111/j.1398-9995.2006.01304.x 17298421

[9] Kurashima K Mukaida N Fujimura M Schroder JM Matsuda T Matsushima K Increase of chemokine levels in sputum precedes exacerbation of acute asthma attacks. J Leukoc Biol (1996) 59:313–6. 10.1002/jlb.59.3.313 8604007

[10] Lamblin C Gosset P Tillie-Leblond I Saulnier F Marquette CH Wallaert B Bronchial neutrophilia in patients with noninfectious status asthmaticus. Am J Respir Crit Care Med (1998) 157:394–402. 10.1164/ajrccm.157.2.97-02099 9476849

[11] Schuh JM Blease K Hogaboam CM CXCR2 is necessary for the development and persistence of chronic fungal asthma in mice. J Immunol (2002) 168:1447–56. 10.4049/jimmunol.168.3.1447 11801688

[12] Todd CM Salter BM Murphy DM Watson RM Howie KJ Milot J The effects of a CXCR1/CXCR2 antagonist on neutrophil migration in mild atopic asthmatic subjects. Pulm Pharmacol Ther (2016) 41:34–9. 10.1016/j.pupt.2016.09.005 27640067

[13] Watz H Uddin M Pedersen F Kirsten A Goldmann T Stellmacher F Effects of the CXCR2 antagonist AZD5069 on lung neutrophil recruitment in asthma. Pulm Pharmacol Ther (2017) 45:121–3. 10.1016/j.pupt.2017.05.012 28549850

[14] Yang T Li Y Lyu Z Huang K Corrigan CJ Ying S Characteristics of Proinflammatory Cytokines and Chemokines in Airways of Asthmatics: Relationships with Disease Severity and Infiltration of Inflammatory Cells. Chin Med J (Engl) (2017) 130:2033–40. 10.4103/0366-6999.213428 PMC5586170 28836545

[15] Wilson MS Wynn TA Pulmonary fibrosis: pathogenesis, etiology and regulation. Mucosal Immunol (2009) 2:103–21. 10.1038/mi.2008.85 PMC2675823 19129758

[16] Car BD Meloni F Luisetti M Semenzato G Gialdroni-Grassi G Walz A Elevated IL-8 and MCP-1 in the bronchoalveolar lavage fluid of patients with idiopathic pulmonary fibrosis and pulmonary sarcoidosis. Am J Respir Crit Care Med (1994) 149:655–9. 10.1164/ajrccm.149.3.8118632 8118632

[17] Carre PC Mortenson RL King TE Jr Noble PW Sable CL Riches DW Increased expression of the interleukin-8 gene by alveolar macrophages in idiopathic pulmonary fibrosis. A potential mechanism for the recruitment and activation of neutrophils in lung fibrosis. J Clin Invest (1991) 88:1802–10. 10.1172/JCI115501 PMC295747 1752942

[18] Bertini R Barcelos LS Beccari AR Cavalieri B Moriconi A Bizzarri C Receptor binding mode and pharmacological characterization of a potent and selective dual CXCR1/CXCR2 non-competitive allosteric inhibitor. Br J Pharmacol (2012) 165:436–54. 10.1111/j.1476-5381.2011.01566.x PMC3268197 21718305

[19] Russo RC Garcia CC Barcelos LS Rachid MA Guabiraba R Roffe E Phosphoinositide 3-kinase gamma plays a critical role in bleomycin-induced pulmonary inflammation and fibrosis in mice. J Leukoc Biol (2011) 89:269–82. 10.1189/jlb.0610346 21048214

[20] Russo RC Guabiraba R Garcia CC Barcelos LS Roffe E Souza AL Role of the chemokine receptor CXCR2 in bleomycin-induced pulmonary inflammation and fibrosis. Am J Respir Cell Mol Biol (2009) 40:410–21. 10.1165/rcmb.2007-0364OC 18836137

[21] Besnard AG Struyf S Guabiraba R Fauconnier L Rouxel N Proost P CXCL6 antibody neutralization prevents lung inflammation and fibrosis in mice in the bleomycin model. J Leukoc Biol (2013) 94:1317–23. 10.1189/jlb.0313140 23975892

[22] Tuder RM Petrache I Pathogenesis of chronic obstructive pulmonary disease. J Clin Invest (2012) 122:2749–55. 10.1172/JCI60324 PMC3408733 22850885

[23] Oostwoud LC Gunasinghe P Seow HJ Ye JM Selemidis S Bozinovski S Apocynin and ebselen reduce influenza A virus-induced lung inflammation in cigarette smoke-exposed mice. Sci Rep (2016) 6:20983. 10.1038/srep20983 26877172 PMC4753462

[24] Jeffery PK Comparison of the structural and inflammatory features of COPD and asthma. Giles F Filley Lecture Chest (2000) 117:251S–60S. 10.1378/chest.117.5_suppl_1.251S 10843939

[25] Qiu Y Zhu J Bandi V Atmar RL Hattotuwa K Guntupalli KK Biopsy neutrophilia, neutrophil chemokine and receptor gene expression in severe exacerbations of chronic obstructive pulmonary disease. Am J Respir Crit Care Med (2003) 168:968–75. 10.1164/rccm.200208-794OC 12857718

[26] Humbles AA Lloyd CM McMillan SJ Friend DS Xanthou G McKenna EE A critical role for eosinophils in allergic airways remodeling. Science (2004) 305:1776–9. 10.1126/science.1100283 15375268

[27] Campa CC Silva RL Margaria JP Pirali T Mattos MS Kraemer LR Inhalation of the prodrug PI3K inhibitor CL27c improves lung function in asthma and fibrosis. Nat Commun (2018) 9:5232. 10.1038/s41467-018-07698-6 30542075 PMC6290777

[28] Kurowska-Stolarska M Kewin P Murphy G Russo RC Stolarski B Garcia CC IL-33 induces antigen-specific IL-5+ T cells and promotes allergic-induced airway inflammation independent of IL-4. J Immunol (2008) 181:4780–90. 10.4049/jimmunol.181.7.4780 18802081

[29] Raemdonck K de Alba J Birrell MA Grace M Maher SA Irvin CG A role for sensory nerves in the late asthmatic response. Thorax (2012) 67:19–25. 10.1136/thoraxjnl-2011-200365 21841185

[30] Dejager L Dendoncker K Eggermont M Souffriau J Van Hauwermeiren F Willart M Neutralizing TNFalpha restores glucocorticoid sensitivity in a mouse model of neutrophilic airway inflammation. Mucosal Immunol (2015) 8:1212–25. 10.1038/mi.2015.12 25760421

[31] Bezerra FS Valenca SS Pires KM Lanzetti M Pimenta WA Schmidt AC Long-term exposure to cigarette smoke impairs lung function and increases HMGB-1 expression in mice. Respir Physiol Neurobiol (2011) 177:120–6. 10.1016/j.resp.2011.03.023 21457800

[32] Garau A Bertini R Mosca M Bizzarri C Anacardio R Triulzi S Development of a systemically-active dual CXCR1/CXCR2 allosteric inhibitor and its efficacy in a model of transient cerebral ischemia in the rat. Eur Cytokine Netw (2006) 17:35–41. 16613761

[33] Russo RC Savino B Mirolo M Buracchi C Germano G Anselmo A The atypical chemokine receptor ACKR2 drives pulmonary fibrosis by tuning influx of CCR2(+) and CCR5(+) IFNgamma-producing gammadeltaT cells in mice. Am J Physiol Lung Cell Mol Physiol (2018) 314:L1010–25. 10.1152/ajplung.00233.2017 29469612

[34] Insuela DB Daleprane JB Coelho LP Silva AR P.M. e Silva MA Carvalho VF Glucagon induces airway smooth muscle relaxation by nitric oxide and prostaglandin E(2). J Endocrinol (2015) 225:205–17. 10.1530/JOE-14-0648 26021821

[35] Draper HH Hadley M Malondialdehyde determination as index of lipid peroxidation. Methods Enzymol (1990) 186:421–31. 10.1016/0076-6879(90)86135-I 2233309

[36] Russo RC Alessandri AL Garcia CC Cordeiro BF Pinho V Cassali GD Therapeutic effects of evasin-1, a chemokine binding protein, in bleomycin-induced pulmonary fibrosis. Am J Respir Cell Mol Biol (2011) 45:72–80. 10.1165/rcmb.2009-0406OC 20833968

[37] Barnes PJ The cytokine network in asthma and chronic obstructive pulmonary disease. J Clin Invest (2008) 118:3546–56. 10.1172/JCI36130 PMC2575722 18982161

[38] Wenzel SE Asthma phenotypes: the evolution from clinical to molecular approaches. Nat Med (2012) 18:716–25. 10.1038/nm.2678 22561835

[39] Robinson DS Hamid Q Ying S Tsicopoulos A Barkans J Bentley AM Predominant TH2-like bronchoalveolar T-lymphocyte population in atopic asthma. N Engl J Med (1992) 326:298–304. 10.1056/NEJM199201303260504 1530827

[40] Anderson GP Endotyping asthma: new insights into key pathogenic mechanisms in a complex, heterogeneous disease. Lancet (2008) 372:1107–19. 10.1016/S0140-6736(08)61452-X 18805339

[41] McKinley L Alcorn JF Peterson A Dupont RB Kapadia S Logar A TH17 cells mediate steroid-resistant airway inflammation and airway hyperresponsiveness in mice. J Immunol (2008) 181:4089–97. 10.4049/jimmunol.181.6.4089 PMC3638757 18768865

[42] Govindaraju V Michoud MC Al-Chalabi M Ferraro P Powell WS Martin JG Interleukin-8: novel roles in human airway smooth muscle cell contraction and migration. Am J Physiol Cell Physiol (2006) 291:C957–65. 10.1152/ajpcell.00451.2005 16822944

[43] Miller AL Strieter RM Gruber AD Ho SB Lukacs NW CXCR2 regulates respiratory syncytial virus-induced airway hyperreactivity and mucus overproduction. J Immunol (2003) 170:3348–56. 10.4049/jimmunol.170.6.3348 12626595

[44] Busse WW Banks-Schlegel S Wenzel SE Pathophysiology of severe asthma. J Allergy Clin Immunol (2000) 106:1033–42. 10.1067/mai.2000.111307 11112883

[45] Manni ML Trudeau JB Scheller EV Mandalapu S Elloso MM Kolls JK The complex relationship between inflammation and lung function in severe asthma. Mucosal Immunol (2014) 7:1186–98. 10.1038/mi.2014.8 PMC4138304 24549277

[46] Shaw DE Berry MA Hargadon B McKenna S Shelley MJ Green RH Association between neutrophilic airway inflammation and airflow limitation in adults with asthma. Chest (2007) 132:1871–5. 10.1378/chest.07-1047 17925424

[47] Barnes PJ New therapies for asthma: is there any progress? Trends Pharmacol Sci (2010) 31:335–43. 10.1016/j.tips.2010.04.009 20554041

[48] Bonecchi R Facchetti F Dusi S Luini W Lissandrini D Simmelink M Induction of functional IL-8 receptors by IL-4 and IL-13 in human monocytes. J Immunol (2000) 164:3862–9. 10.4049/jimmunol.164.7.3862 10725748

[49] Hoth JJ Wells JD Hiltbold EM McCall CE Yoza BK Mechanism of neutrophil recruitment to the lung after pulmonary contusion. Shock (2011) 35:604–9. 10.1097/SHK.0b013e3182144a50 PMC3108153 21330942

[50] Wedzicha JA Seemungal TA MacCallum PK Paul EA Donaldson GC Bhowmik A Acute exacerbations of chronic obstructive pulmonary disease are accompanied by elevations of plasma fibrinogen and serum IL-6 levels. Thromb Haemost (2000) 84:210–5. 10.1055/s-0037-1613998 10959691

[51] Kaplanski G Marin V Montero-Julian F Mantovani A Farnarier C IL-6: a regulator of the transition from neutrophil to monocyte recruitment during inflammation. Trends Immunol (2003) 24:25–9. 10.1016/S1471-4906(02)00013-3 12495721

[52] Hashimoto M Tanaka H Abe S Quantitative analysis of bronchial wall vascularity in the medium and small airways of patients with asthma and COPD. Chest (2005) 127:965–72. 10.1378/chest.127.3.965 15764783

[53] Hallgren J Jones TG Abonia JP Xing W Humbles A Austen KF Pulmonary CXCR2 regulates VCAM-1 and antigen-induced recruitment of mast cell progenitors. Proc Natl Acad Sci U.S.A. (2007) 104:20478–83. 10.1073/pnas.0709651104 PMC2154456 18077323

[54] Chapman RW Minnicozzi M Celly CS Phillips JE Kung TT Hipkin RW A novel, orally active CXCR1/2 receptor antagonist, Sch527123, inhibits neutrophil recruitment, mucus production, and goblet cell hyperplasia in animal models of pulmonary inflammation. J Pharmacol Exp Ther (2007) 322:486–93. 10.1124/jpet.106.119040 17496165

[55] Govindaraju V Michoud MC Ferraro P Arkinson J Safka K Valderrama-Carvajal H The effects of interleukin-8 on airway smooth muscle contraction in cystic fibrosis. Respir Res (2008) 9:76. 10.1186/1465-9921-9-76 19046427 PMC2633308

[56] Halwani R Al-Abri J Beland M Al-Jahdali H Halayko AJ Lee TH CC and CXC chemokines induce airway smooth muscle proliferation and survival. J Immunol (2011) 186:4156–63. 10.4049/jimmunol.1001210 21368236

[57] Nirodi CS Devalaraja R Nanney LB Arrindell S Russell S Trupin J Chemokine and chemokine receptor expression in keloid and normal fibroblasts. Wound Repair Regener (2000) 8:371–82. 10.1111/j.1524-475X.2000.00371.x PMC3140346 11115149

[58] Nair P Gaga M Zervas E Alagha K Hargreave FE O’Byrne PM Safety and efficacy of a CXCR2 antagonist in patients with severe asthma and sputum neutrophils: a randomized, placebo-controlled clinical trial. Clin Exp Allergy (2012) 42:1097–103. 10.1111/j.1365-2222.2012.04014.x 22702508

[59] Roberts G Chen S Yates P Madan A Walker J Washburn ML Randomized, Double-Blind, Placebo-Controlled Study of the Safety, Tolerability, and Clinical Effect of Danirixin in Adults With Acute, Uncomplicated Influenza. Open Forum Infect Dis (2019) 6(4):ofz072. 10.1093/ofid/ofz072 31024969 PMC6476494

[60] Rennard SI Dale DC Donohue JF Kanniess F Magnussen H Sutherland ER CXCR2 Antagonist MK-7123. A Phase 2 Proof-of-Concept Trial for Chronic Obstructive Pulmonary Disease. Am J Respir Crit Care Med (2015) 191:1001–11. 10.1164/rccm.201405-0992OC 25695403

[61] Joseph JP Reyes E Guzman J O’Doherty J McConkey H Arri S CXCR2 Inhibition - a novel approach to treating CoronAry heart DiseAse (CICADA): study protocol for a randomised controlled trial. Trials (2017) 18:473. 10.1186/s13063-017-2210-2 29020983 PMC5637263

[62] Eash KJ Greenbaum AM Gopalan PK Link DC CXCR2 and CXCR4 antagonistically regulate neutrophil trafficking from murine bone marrow. J Clin Invest (2010) 120:2423–31. 10.1172/JCI41649 PMC2898597 20516641

[63] Lazaar AL Miller BE Tabberer M Yonchuk J Leidy N Ambery C Effect of the CXCR2 antagonist danirixin on symptoms and health status in COPD. Eur Respir J (2018) 52:1801020. 10.1183/13993003.01020-2018 30139779

[64] Barnes PJ Chronic obstructive pulmonary disease. N Engl J Med (2000) 343:269–80. 10.1056/NEJM200007273430407 10911010

[65] Ko FW Chan KP Hui DS Goddard JR Shaw JG Reid DW Acute exacerbation of COPD. Respirology (2016) 21:1152–65. 10.1111/resp.12780 PMC7169165 27028990

