# Supplementary figures and images for: CXCR1 and CXCR2 Inhibition by Ladarixin Improves Neutrophil-Dependent Airway Inflammation in Mice
Source: Front Immunol. 2020 Oct 2;11:566953. doi: 10.3389/fimmu.2020.566953 (PMC7566412; doi:10.3389/fimmu.2020.566953)

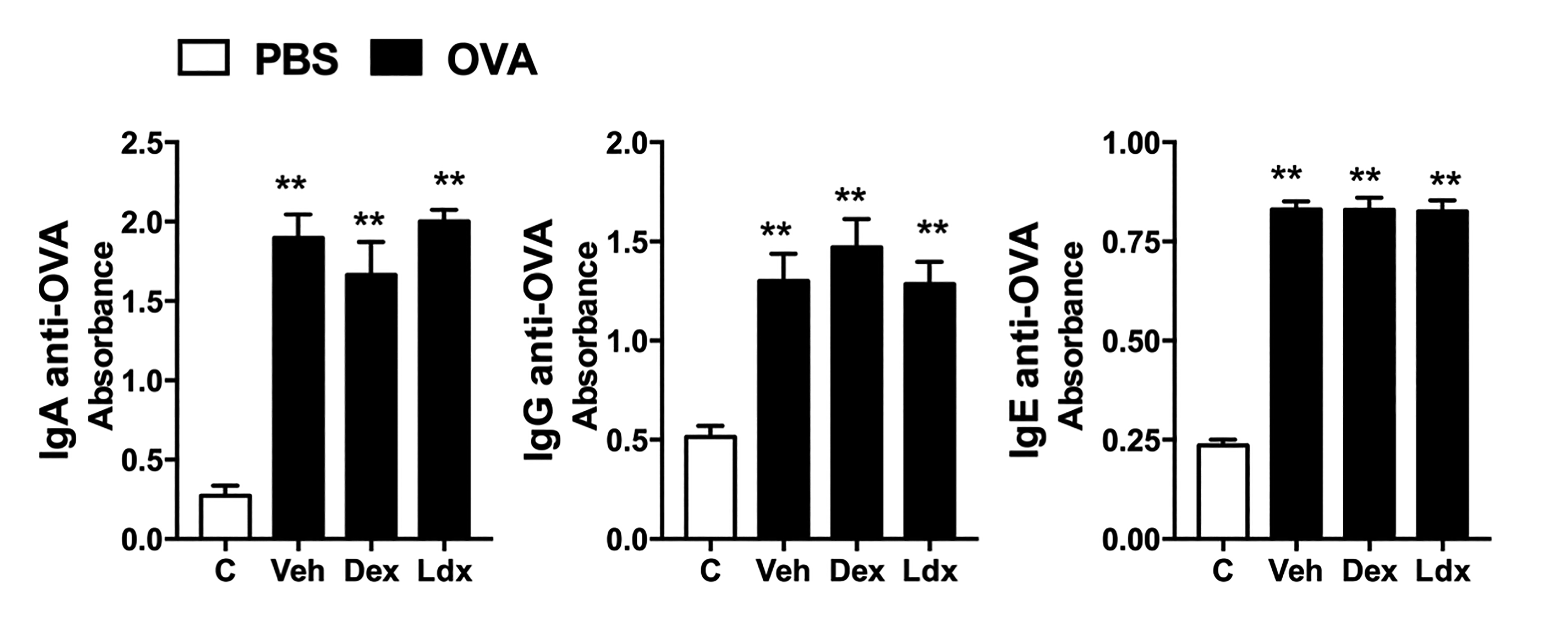

Supplement: Supplementary Figure 1 — Mice atopy induced by OVA challenge. IgA, IgG and IgE antibodies levels were measured by ELISA. n = 8 for each group, * for P < 0.05; ** for P < 0.01. [file Image_1.jpeg]

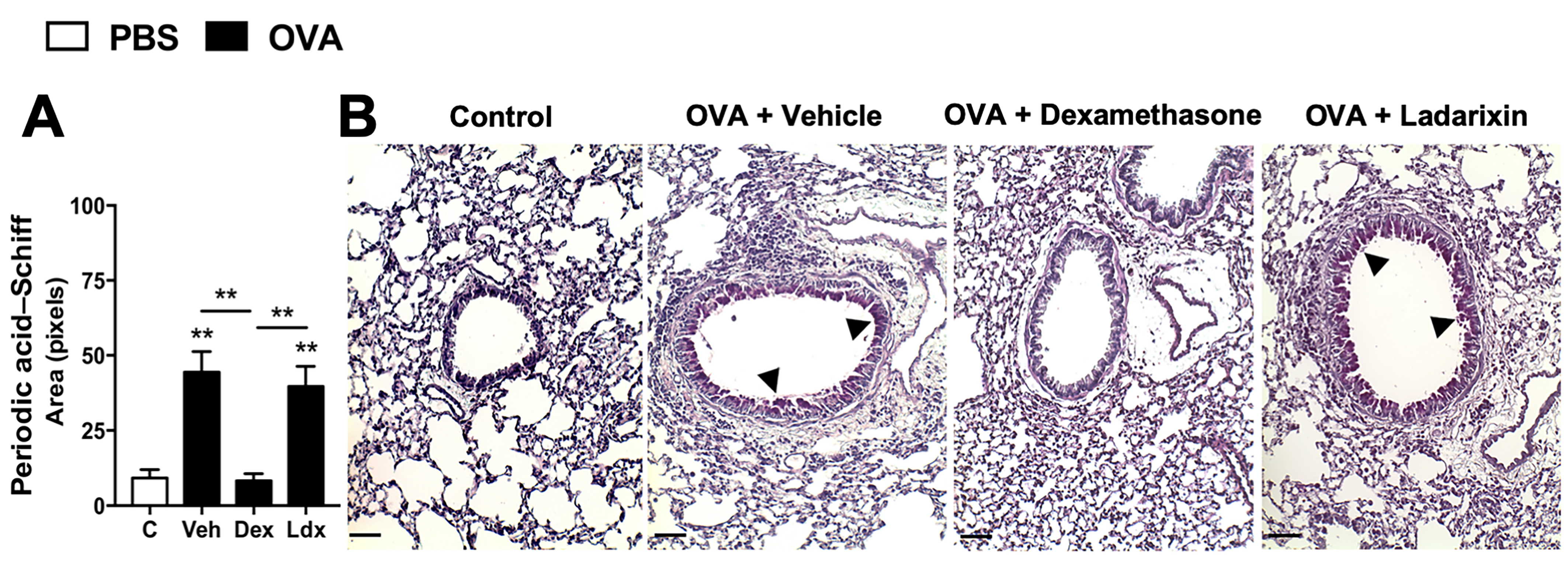

Supplement: Supplementary Figure 2 — Ladarixin treatment did not reduces mucus overproduction. Mucus production was evaluated after four daily challenges with OVA. (A) Histogram showing area positive for mucus, which indicates goblet cell hypertrophy. (B) Lung histological sections (Periodic acid-schiff dye). Arrows head indicate goblet cells hypertrophy and mucus overproduction. n = 8 for each group, ** for P < 0.01. [file Image_2.jpeg]

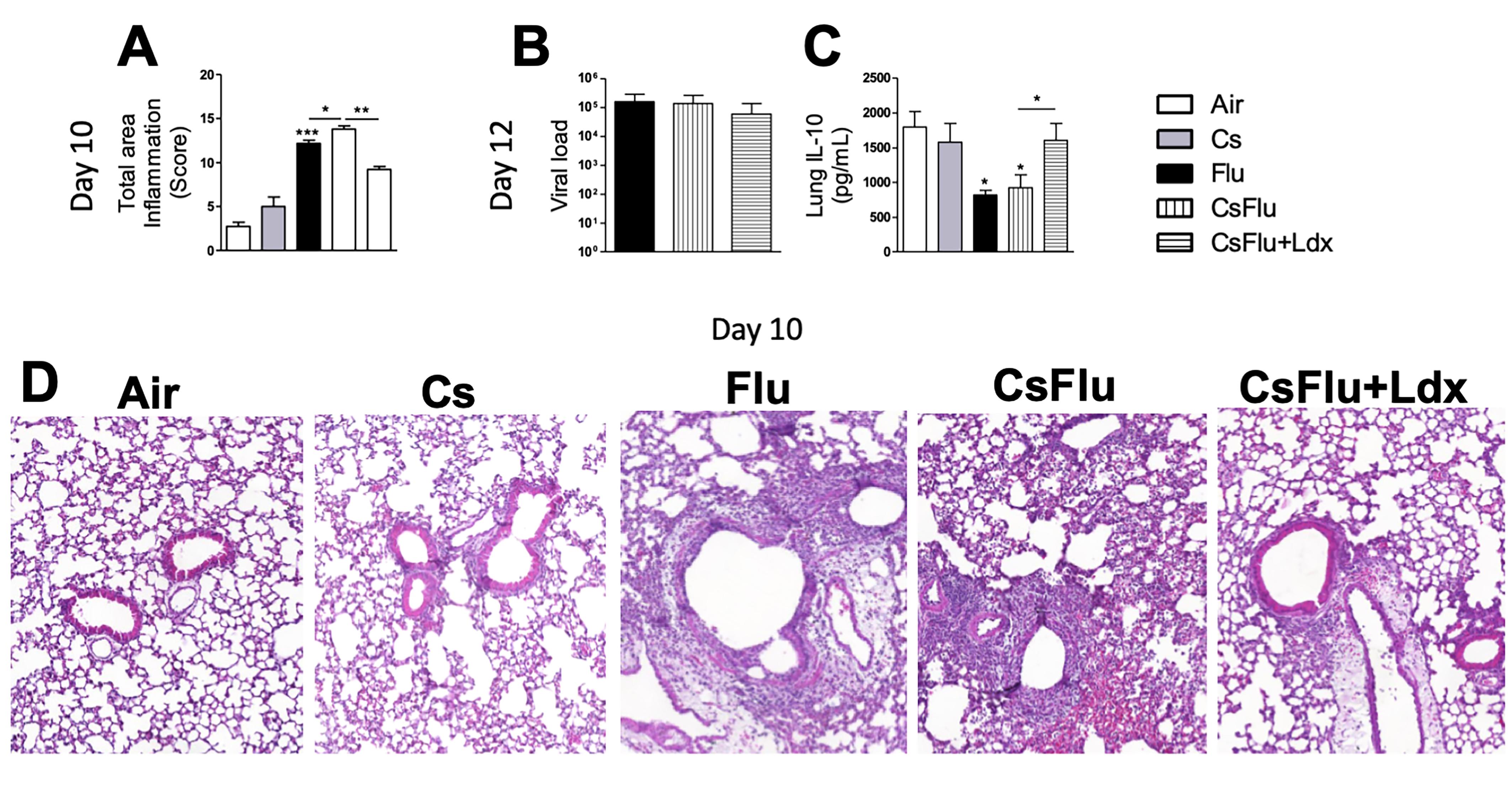

Supplement: Supplementary Figure 3 — Ladarixin effect over lung histological sections, viral titer and IL-10 production in lung tissue. (A) Inflammation score, derived from histological analyses from lung tissue, obtained at day 10 of Cs exposure. (B) Lung viral titer from infected mice groups on day 12 of assay. (C) IL-10 levels in lung tissue of mice obtained on day 12 of assay. (D) Representative histological sections of lung (H&E dye) for each group obtained at day 10 of Cs exposure. n = 6 for each group, * represents significant differences compared to Air group except when indicated with a solid line * for P < 0.05 and ** for P < 0.01. [file Image_3.jpeg]
